# Identified endoplasmic reticulum stress-related molecular cluster and immune characterization in endometriosis

**DOI:** 10.1038/s41598-025-22400-9

**Published:** 2025-11-04

**Authors:** Erqing Huang, Ling Zhang, Jie Lou, Xiaoli Wang, Lijuan Chen

**Affiliations:** 1 Department of Obstetrics and Gynecology, Union Hospital, Tongji Medical College, Huazhong University of Science and Technology, Wuhan, 430022 China; 2 Department of Radiology, Union Hospital, Tongji Medical College, Huazhong University of Science and Technology, Wuhan, 430022 China

**Keywords:** Endometriosis, Endoplasmic reticulum stress, Bioinformatic analysis, Immune infiltration, Stromal cells, Cell biology, Computational biology and bioinformatics, Biomarkers

## Abstract

Endometriosis is a common disease among women of childbearing age, and endoplasmic reticulum stress (ERS), a response involved in regulating protein homeostasis, has been linked to its pathogenesis. To identify ERS-related hub genes, this study sequentially employed differential expression analysis, weighted gene co-expression network analysis (WGCNA), protein–protein interaction (PPI) network construction, and three machine learning algorithms. These methods led to the identification of four hub genes: Von Willebrand factor (VWF), vascular cell adhesion molecule 1 (VCAM1), endothelial PAS domain protein 1 (EPAS1), and coagulation factor VIII (F8). Unsupervised cluster analysis was conducted to categorize samples into ERS clusters, and the CIBERSORT algorithm was used to calculate immune infiltration scores, revealing two stable clusters. Cluster B was defined as “immune-enriched” with significantly higher immune scores, while Cluster A was “less immune-enriched”. Functional enrichment analysis of differentially expressed genes (DEGs) between the clusters highlighted cell adhesion and regulation of immune cell activation as key to cluster-specific phenotypes. A diagnostic model built with the four hub genes showed robust utility via validation curves, confirming their clinical relevance. DEGs from each cluster were screened in the Connectivity Map database to identify cluster-specific therapeutic agents. RT-qPCR and immunohistochemistry (IHC) validated that both mRNA and protein levels of the four hub genes were elevated in endometriosis tissues, supporting the bioinformatics findings. Overall, this study links ERS-related hub genes to endometriosis subtyping, immune infiltration, and diagnostics, providing a basis for personalized treatments and a potential clinical tool.

**Supplementary Information:**

The online version contains supplementary material available at 10.1038/s41598-025-22400-9.

## Introduction

Endometriosis (EMS) is defined as the presence of endometrial tissue outside the uterus^1^. Approximately 10% of women of reproductive age globally are estimated to be afflicted by this ailment^2^. The main manifestations of endometriosis encompass dysmenorrhea, chronic pelvic pain, infertility, painful intercourse. If the ectopic tissue affects bladder and rectum, it may also lead to corresponding dysfunctions in the urinary and digestive systems, such as constipation and hematuria^3–5^. Currently, laparoscopic surgery to remove the lesions is still the gold standard in the treatment of endometriosis therapy. Nonetheless, the disorder is extremely prone to recurrence, thereby rendering long-term management a formidable challenge.

The endoplasmic reticulum serves as a crucial organelle within organisms, facilitating the synthesis and modification of proteins. Proper protein folding processes are pivotal in dictating cellular outcomes^6^. The unfolded protein response (UPR) is initiated by transmembrane proteoceptors including IRE1α, PERK, and ATF6, whereby the chaperone GRP78/BiP typically binds to these receptors. When exogenous or endogenous endoplasmic reticulum stressors result in misfolded endoplasmic reticulum proteins, BiP dissociates from the receptor and binds to unfolded proteins, which activates the UPR^7^. The UPR initially responds to adaptive endoplasmic reticulum stress, however, under irremediable endoplasmic reticulum stress, the UPR can trigger pro-inflammatory and pro-death signals. Emerging evidence indicates that multiple endoplasmic reticulum stress sensors are activated within the tumor immune microenvironment, and the protective function of innate immune cells is disrupted by endoplasmic reticulum stress, and promote tumor cell proliferation and progression^8–10^. Multiple studies have demonstrated that induced endoplasmic reticulum stress can impede the proliferation and invasion of endometriosis lesions by modulating signaling pathways such as Akt/mTOR, MAPK/ERK, and NF-κB^11–15^. For instance, a study on the first-line therapeutic agent for endometriosis, dienogest, confirmed that this fourth-generation synthetic progestin upregulates endoplasmic reticulum stress in endometriotic stromal cells. This augmentation results in the upregulation of CHOP, thereby promoting apoptosis, restraining cell proliferation and invasion, and mitigating lesion progression in endometriosis^16^. Another study on ovarian endometriosis granulosa cells found that endoplasmic reticulum stress mediated apoptosis and subsequent ovarian dysfunction in patients’ granulosa cells through caspase-3 and caspase-8^17^. These findings underscore the significant implication of endoplasmic reticulum stress in the pathogenesis of endometriosis. Nevertheless, the specific molecular subtypes of the disease linked to endoplasmic reticulum stress, as well as their immunological attributes, remain to be elucidated.

Although endometriosis is known as a benign disease, it has demonstrated characteristics of angiogenesis, tissue invasion, cell implantation to distant organs and malignant transformation. Activation of tumor-like complement pathways and humoral immunity is present in some patients with endometriosis, perhaps favoring immunosuppression and neovascularization. It has been demonstrated that infiltration of CD3 + CD4 + and CD3 + CD8 + T cells may be indicative of malignant transformation in endometriosis^18^. We have mentioned before that ER stress is involved in the regulation of the immune microenvironment in other diseases, therefore, we would like to know the effect of endoplasmic reticulum stress on the immune microenvironment of endometriosis, and whether endoplasmic reticulum stress is related to the pathophysiology properties of the disease, such as angiogenesis, invasion, adhesion and metastasis.

Since limited research has elucidated the specific roles of ERS in endometriosis through bioinformatic approaches, we performed a bioinformatic analysis to establish an ERS- and immune-associated subtyping model, aiming to facilitate prospective clinical applications in the diagnosis and management of endometriosis. Our study established a diagnostic model for endometriosis consisting of four endoplasmic reticulum stress-related genes, which are VWF, F8, EPAS1, and VCAM1.VWF is often regarded as a marker of vascular endothelial damage, and endoplasmic reticulum stress levels were increased in a mouse model of systemic lupus erythematosus-associated diffuse alveolar hemorrhage (DAH) along with the levels of VWF, suggesting that VWF may be a target of endoplasmic reticulum stress leading to vascular endothelial dysfunction-associated diseases^19^. F8 is an important component of the coagulation system, and its deficiency can lead to hemophilia A. Expression of FVIII activates the endoplasmic reticulum (ER) stress response, leading to oxidative stress and inducing apoptosis^20^. EPAS1 alias HIF-2α, HIF-2α-deficient hematopoietic stem and progenitor cells show increased ROS production is increased and activation of the unfolded protein response (UPR) pathway triggers apoptosis^21^. VCAM1 is an inflammatory factor and inhibition of endoplasmic reticulum stress reduces the production of VCAM1 and IL-6^22^. Therefore, exploring the relationship between endoplasmic reticulum stress-related genes (ERSRGs) and endometriosis may help clarify the endometriosis heterogeneity at a molecular level.

## Materials and methods

### Patients and sample collection

From February 2023 through December 2023, we prospectively collected normal control, eutopic and ectopic endometrial samples. This research was approved by the Ethics Committee of Union Hospital, Tongji Medical College, Huazhong University of Science and Technology (IRB: S0496). Written informed consent was obtained from patients before the collection of human tissues, in accordance with the guidelines of the Declaration of Helsinki. Detailed patient information is provided in the Supplement material 1.

Normal endometrial tissue samples were obtained from patients without endometriosis who underwent hysteroscopy and endometrial biopsy prior to pathological diagnosis, which confirmed as normal endometrium post-procedure (n = 10). Furthermore, ectopic (n = 10) and eutopic endometrial samples (n = 7) were sourced from individuals diagnosed with stage III or IV endometriosis^23^. All the collected endometrial tissues were diagnosed as proliferative endometrium after pathological histological diagnosis. All menstrual cycles were normal, non-pregnant or non-lactation, and no hormonal medication was taken 6 months before the operation, and no obvious medical and surgical diseases and complications were found.

### Data collection

GSE7305 (https://www.ncbi.nlm.nih.gov/geo/query/acc.cgi?acc=GSE7305) and GSE11691(https://www.ncbi.nlm.nih.gov/geo/query/acc.cgi?acc=GSE11691) datasets were downloaded from the Gene Expression Omnibus (GEO) database as training set by using the R package “GEOquery”. Details of datasets are listed in Table 1. GSE11691 was in GPL96 platform, which consisted 9 peritoneal endometriosis and 9 normal endometrial tissue samples (Control samples). GSE7305 was in GPL570 platform, which consisted 10 ovarian endometriosis and 10 normal endometrial tissue samples (Control samples). Then, we combined two datasets by using the “limma” package, the batch effect of the original gene expression landscapes in two datasets was eliminated using the comBat function based on the “sva” package. A total of 19 endometriosis tissue samples and 19 normal endometrium tissue samples with complete mRNA expression data and corresponding clinical materials were selected for subsequent analysis.

**Table 1:** Information on the GEO dataset used in this study.

Sample	Platforms	Use in this article	Normal endometrium tissue	Eutopic endometrium tissue	Ovarian endometriosis tissue	Peritoneal endometriosis tissue	All
GSE7305^24^	GPL570	Training set	10	0	10	0	20
GSE11691^25^	GPL96	Training set	9	0	0	9	18
GSE51981^26^	GPL570	Training set	71	77	0	0	148
GSE23339^27^	GPL6102	Test set	9	0	10	0	19
GSE25628^28^	GPL571	Test set	6	8	8	0	22

### Identification of differentially expressed endoplasmic reticulum stress-related genes in endometriosis

We extracted 1350 endoplasmic reticulum stress-related genes (ERSRGs) from previous articles, gene list are provided in Supplement Material 2. Genes with adjusted *p*-values (Benjamini–Hochberg method, FDR < 0.01) and |log_2_FC|> 0.5 were considered differentially expressed genes (DEGs) by “limma” R package. The results were visualized using “ggplot2” and “ComplexHeatmap” packages.

### Analyzing and comparing immune infiltrating cells between endometriosis and normal endometrium

CIBERSORT algorithm (https:/cibersort.stanford.edu/) was applied to estimate the abundance of 21 immunocyte subtypes in endometriosis and normal samples, which was performed on R software. The ESTIMATE algorithm was first used for tumor-related immune cell infiltration assessment. It first uses gene expression profiling data to estimate the ratio of disease tissue cells to normal tissue cells. Then, it estimates the abundance of immune cells in disease tissue by comparing the variability of gene expression. Finally, the immune infiltration score is calculated by combining the estimated disease tissue cell proportion and the abundance of immune cells. ESTIMATE algorithm was adopted to measure immunocyte infiltration degree (ImmuneScore) in these samples. The Wilcoxon test is a nonparametric hypothesis test for comparing the difference between two related totals, which is applied when the samples come from the same total but do not obey a normal distribution. Student’s t-test was applied to verify the differences between the two groups, and results were presented using the “ggboxplot”, “pheatmap” and “corrplot” packages. Single sample gene set enrichment analysis (ssGSEA) was performed to determine enrichment scores for each coupling of a sample and immune reaction gene sets in the ImmPort database. The package “GSVA” was used for the ssGSEA analysis.

### Construction of weighted gene coexpression networks (WGCNA)

WGCNA (Weighted correlation network analysis) is a systems biology method used to characterize patterns of gene association between samples, which can be used to identify highly synergistic sets of genes and to identify candidate biomarker genes or therapeutic targets based on gene-set introgression and gene-set-phenotype associations. The identification of candidate biomarker genes or therapeutic targets based on gene set endlinkage and gene set-phenotype association. The package “WGCNA” was used for constructing the co-expression network of all genes between the 19 endometriosis and 19 normal samples. We obtained the difference between gene modules and endometriosis related modules hub genes. Hierarchical clustering was conducted on these samples to detect the outliers and remove the abnormal samples. The optimal power value was selected to transform the gene expression matrix into a weighted adjacency matrix, which was further transformed into a topological overlap matrix (TOM). Finally, hierarchical clustering was used to classify strongly correlated sub-networks (co-expressed gene modules) based on 1-TOM. More detailed of WGCNA are contained in Supplement Material 2.Correlations between modules and traits were then calculated using the WGCNA package. Modules with high correlation coefficients were considered as candidates related to endometriosis and selected for subsequent analyses. With the candidate module selected, we defined |MM| (|Module membership|) > 0.8 and |GS| (|gene significance|) > 0.5 as the screening criteria for filtering key genes in the candidate module. The intersection of differentially expressed endometriosis genes related to ERS and hub genes in key modules were performed using the “jvenn” online website (https://jvenn.toulouse.inrae.fr/app/example.html).

### Identification of ERS-related key genes in endometriosis and chromosome location

Based on above genes obtained intersecting genes, we constructed of protein–protein interaction (PPI) networks in STRING database (http: //string-db. org/). Subsequently, Cytoscape software was used to visualize the PPI network. Maximal Clique Centrality (MCC) algorithm evaluates the centrality of a node by calculating the maximum number of clusters to which the node belongs. A maximal clique is a subgraph in which every node is directly connected to all other nodes. With this algorithm we can find the core proteins in the PPI network. Then, we used the MCC algorithm in the cytoHubba (a Cytoscape plugin) to screen the key genes with high connectivity in PPI networks. Genomic locations of these key genes in chromosomes were demonstrated using “RCircos” package. Then, these key genes were processed with three machine learning algorithms. Least Absolute Shrinkage and Selection Operator (LASSO) to identify the genes by using the “glmnet” package. Random Forest Algorithm Analysis (RF) was conducted by the “randomForest” package. Meanwhile, a support vector machine-recursive feature elimination (SVM-RFE) model was established with a “SVM” package. Genes converged by three machine learning methods were confirmed as the hub ERS-related genes in endometriosis. The diagnostic and clustering discriminative ability of these selected hub genes was assessed through receiver operating characteristic (ROC) curves.

### ROC curve analysis

We chose GSE23339 and GSE25628 dataset as testing sets, performed receiver operating characteristic (ROC) curve analysis on each screened hub ERS-related genes to verify its accuracy. The “pROC” package was used for ROC curve analysis. The hub genes with average AUC > 0.75 in both training and testing set were considered useful for EMS diagnosis.

### Unsupervised clustering of hub ERSRGs in endometriosis

According to the expression level of hub ERSRGs, an unsupervised clustering analysis was carried out to classify 77 endometriosis samples of GSE51981 into distinct clusters by using the R package of “ConsensusClusterPlus”. The optimal number of classifications was determined by the cumulative distribution function (CDF) curves, consistency clustering score of each cluster and consensus clustering plot. We used Principal Component Analysis (PCA) to verify the ERS gene expression patterns. Besides, we calculate the “ERSscore” and “ImmuneScore” in distinct ERS cluster. We can understand these clusters as endoplasmic reticulum stress subtypes with different levels of endoplasmic reticulum stress. Those with ERS scores greater than zero were categorized as “high ERSscore subtype” and those less than zero were categorized as “low ERSscore subtype”. The DEGs between the two clusters were identified by the “limma” package (adjusted *p* < 0.05, |log_2_FC|> 1).

### Functional enrichment analysis and identification of potential drugs

Based on the cluster DEGs, the Gene Ontology (GO) and Kyoto Encyclopedia of Genes and Genomes (KEGG) pathway enrichment analyses between the two subgroups were performed using the “clusterProfiler” package^29,30^. Gene set enrichment analysis (GSEA) is used to evaluate the correlation of cluster DEGs in pre-defined gene set with each ERS cluster. The “c7.all.v2023.1.Hs. symbols” and “c2. kegg. immune. v2023.1.Hs. symbols” gene sets in the MSigDB database were subjected to GSEA using the “clusterProfiler” package. *p*-value < 0.05 was considered statistically significant. For each ERS cluster, we uploaded the cluster DEGs to the connective map (CMap) database and then applied enrichment analysis to pull out significantly enriched drugs. We used filter (with *p* < 0.05) to identify top 60 compounds per ERS cluster that are predicted to treat EMS.

### Clinical trait analysis and diagnostic model construction

Besides, the R package “ggalluvial” constructed a Sankey diagram for demonstrating the distribution trend of ERS cluster, endometriosis severity and ERS score type. Each row represents a feature variable, different color represents different typing or clinical trait, lines represent the distribution of the same sample in different feature variables. Based on above hub ERSRGs obtained from intersection of machine learning methods, we built a nomograph using the “rms” and “rmda” package and evaluated its predictive power using the calibration curve to predict the probability of EMS.

### Immunohistochemistry (IHC) and image analysis

The immunohistochemical staining of paraffin-embedded sections from all samples underwent morphological screening examination and was conducted following the established protocol previously described in the literature^31^. Briefly, the sections were stained with the primary antibody against F8 (1:500, affinity, USA), VCAM1(1:200, Proteintech, Wuhan, China), VWF (1:400, Proteintech, Wuhan, China), and EPAS1(1:50, Proteintech, Wuhan, China) and then scanned with a digital scanner. The intensity of staining was quantified using the ImageJ software. First set all the images to a uniform size and convert the images to RGB-stack format. After the above operation, a scroll bar appears at the bottom of the picture, the left, center and right files correspond to red, green and blue respectively, and the picture with the highest match to the positive signal is selected for subsequent analysis. Then, mark the stained area and set the measurement parameters in the pop-up measurement parameter setting option box. Finally, measure the area share of the positive area and record the accumulated optical density value IntDen (Integrated option density, IOD). IOD value is proportional to the total amount of the target protein, IOD value divided by the area of the target protein distribution area (IntDen/Area), to get the average optical density value AOD (Aver-ageOptical Density), through the AOD value as a statistical graph, can compare the differences in protein expression. Formula: AOD (%Area) = IOD/Area, % Area is the result.

### Quantitative real-time PCR (qRT-PCR)

Total RNA was isolated from ovarian endometriosis, eutopic endometrium and normal control endometrium. We used Trizol (Yeasen, Shanghai, China) to isolate RNA from tissues according to the manufacturer’s instructions. Then, one microgram of RNA was reversely transcribed into cDNA using BeyoRT™ III cDNA First Strand cDNA Synthesis Kit (Beyotime, Shanghai, China). Amplification was performed using gene-specific primers (Qingke, Beijing, China) and SYBR Green qPCR Mix 2 × (Beyotime, Shanghai, China) on a qRT-PCR device (ABI StepOnePlus, America). GAPDH was used as an internal control. The relative expression of the genes was calculated using the 2^–ΔΔCT^ method. The primers are shown in Table 2.

**Table 2:** qRT-PCR primers.

Genes	Forward primers	Reverse primers
EPAS1	GGCTGTGTCTGAGAAGAGTAACT	TCCCGAAATCCAGAGAGATGATG
F8	TGGAGTTGATGGGCTGTGATTTA	CCAGGTGGCAAACATATTGGTAAA
VCAM1	CGGAGACAGGAGACACAGTACTA	GCACGAGAAGCTCAGGAGAAA
VWF	GGGAAGACTGTGATGATCGATGT	GCAAACATCTCCCACAACATTCA
GAPDH	GGAGTCCACTGGCGTCTTCA	GTCATGAGTCCTTCCACGATACC

### Statistical analysis

All statistical analyses were performed using the R programming software (Version R4.3.1). Comparisons between two groups were performed using the t-test. Spearman test was utilized for correlation analysis. A difference of *p* < 0.05 indicated statistical significance unless specified otherwise. (∗ ∗  ∗ represents *p* < 0.001, **represents *p* < 0.01, and * represents *p* < 0.05).

## Results

### The landscape of different expressed ERSRGs and immune cell characteristics between EMS and normal samples

**Fig. 1:**
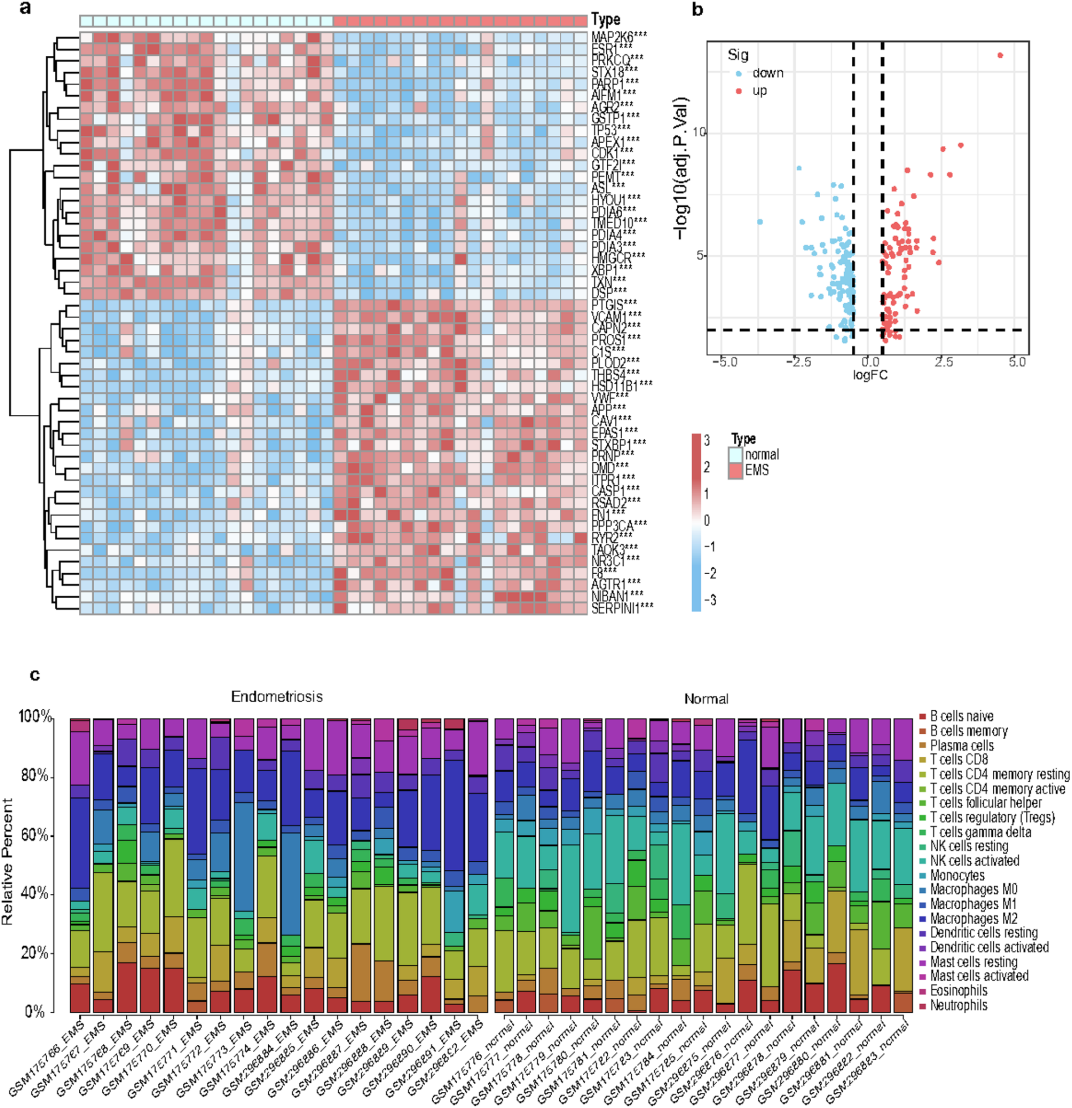
The expression pattern and immune characteristics of the ERSRGs in EMS. (**a**) Heatmap of the top fifty differentially expressed ERSRGs. (**b**) The volcano plot of the 209 different expressed ERSRGs in EMS and normal samples. (**c**) Bar plot of the relative proportion of 21 infiltrated immune cells in EMS and normal sample. ERSRG, endoplasmic reticulum stress-related genes; EMS, endometriosis.

After removing batch effect by comBat, which can use either parametric or non-parametric empirical Bayes frameworks for adjusting data for batch effects, then, differential gene expression analysis was performed using combined dataset of GSE7305 and GSE11691. We totally gained 1350 ERSRGs from published articles, among which 1180 ERSRGs are included in the combined dataset. Heatmaps of top 50 ERS-related DEGs are shown (Fig. 1a), the DEGs exhibit significantly different expression patterns between the EMS and normal samples. A total of 209 ERS-related DEGs (|log_2_FC|> 0.5 and *p* < 0.01) were obtained, including 92 up-regulated and 117 down-regulated genes (Fig. 1b).

**Fig. 2:**
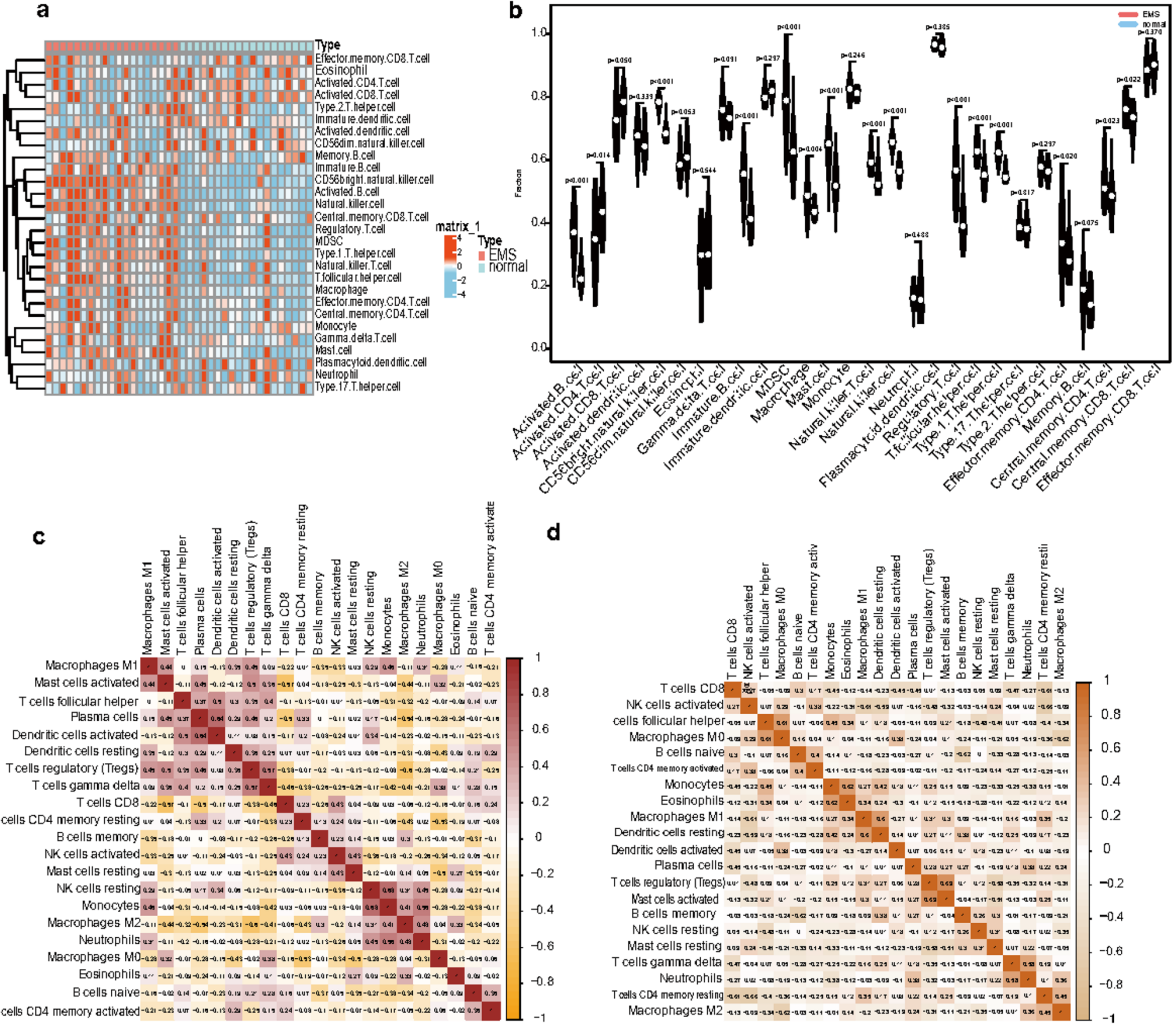
(**a**) Distribution of 28 immune cells and their subtypes in EMS and normal samples. (**b**) Violin plot showing differential infiltration of the 28 immune cell populations. (**c**,**d**) Correlation heatmap of 21 immune cell types in EMS (**c**) and normal (**d**) samples. *p < 0.05, **p < 0.01, ∗  ∗  ∗ *p* < 0.001. EMS, endometriosis.

The proportion of different infiltrating immune cell types between the EMS and normal groups was evaluated using the CIBERSORT algorithm (Fig. 1c). After removing populations with a sum of immune abundance value zero, Wilcoxon test was used to assess the enrichment scores representing immunocyte abundance and immune response activity between EMS and normal samples. As can be concluded from the Fig. 2a, the vast majority of immune cells had higher levels of infiltration in endometriosis samples than in normal control samples, suggesting that EMS is a disease closely related to immune dysregulation, which is consistent with previous findings.

In comparison to normal endometrium, activated B cell, CD56bright natural killer cell, immature B cell, myeloid-derived suppressor cell (MDSC), macrophage, mast cell, natural killer T cell, natural killer cell, regulatory T cell, T follicular helper cell, type1 T helper cell, effector memory CD4 T cell, central memory CD4 T cell, central memory CD8 T cell were found to have higher level of infiltration in EMS (Fig. 2b).

Besides, we conducted the correlation analysis between 21 immunocyte subtypes. We found that dendritic cells activated and plasma cells were the most positive relevant immune cells in EMS samples (r = 0.64), monocytes and eosinophils were the most positive relevant immune cells in normal samples (r = 0.62), which may indicate that these immunocytes work together (Fig. 2c,d).

### Identification of key modules associated with the ERS through WGCNA

**Fig. 3:**
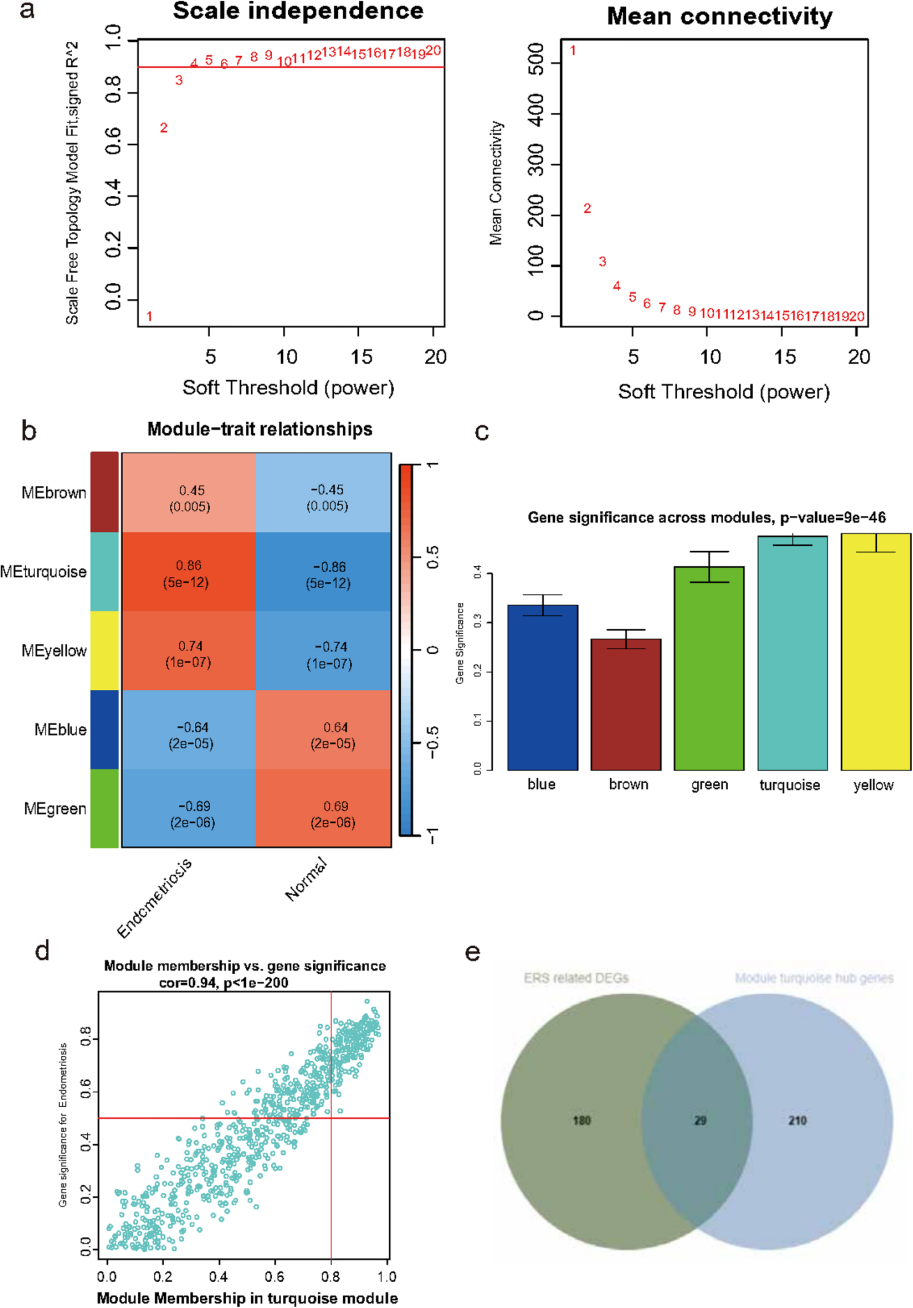
WGCNA finds characteristic EMS genes. (**a**) Analysis of the scale-free ft index and analysis of the mean connectivity for various soft-thresholding powers. (**b**) Correlation between the different modules in EMS and normal sample. (**c**) Bar plot of the importance of gene modules. (**d**) Gene significance (GS) scatter plot of EMS and module member (MM) in the turquoise module. (**e**) Venn diagram showing the intersection of WGCNA turquoise module key genes and ERS related DEGs in EMS. ERS, endoplasmic reticulum stress-related genes; EMS, endometriosis; WGCNA, Weighted Correlation Network Analysis.

In this study, we used WGCNA to cluster highly correlated genes associated with endometriosis. A dendrogram of samples (GSE51981) with clinical trait was clustered using the average linkage method and Pearson’s correlation method. The soft-threshold power was set to 3 to ensure a scale-free network (Fig. 3a). Five co-expression modules were identified through WGCNA analysis. The turquoise module showed highly positively correlation with EMS mRNA (r = 0.86, *p* = 5e-12) (Fig. 3b). Module importance scores also indicate that the turquoise module is the one to focus on (Fig. 3c). Next, the turquoise module was selected as key modules relevant to EMS for further analysis. In Fig. 3F, the significant correlations between gene significance (GS) and module membership (MM) were presented in the turquoise module, 239 module hub genes were found in the two modules by GS > 0.5 and MM > 0.8 (Fig. 3d). Hub genes of module turquoise were intersected with 209 different expressed ERSRGs. There were 29 intersecting genes in the Venn diagram (Fig. 3e).

### Identification of hub ERSRGs in endometriosis

**Fig. 4:**
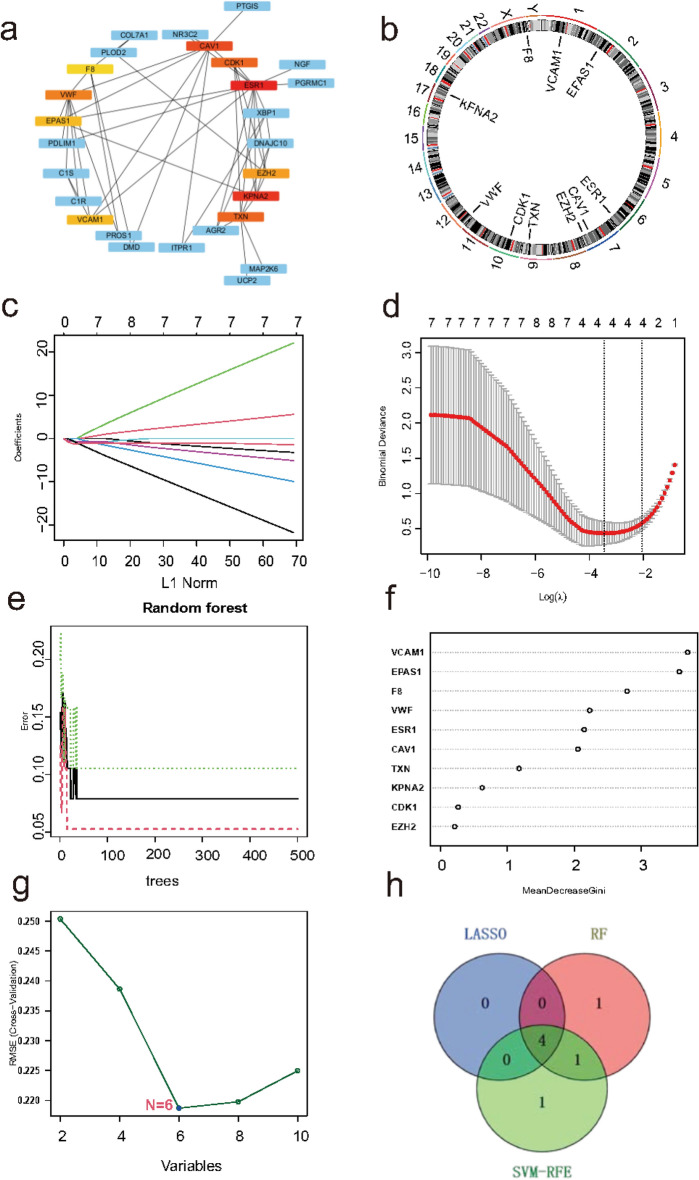
PPI Machine learning arthrograms find hub ERSRGs in EMS. (**a**) PPI network of 29 genes. Top 10 differentially expressed ERSRGs were in red, yellow or orange color. (**b**) The location of top 10 differentially expressed ERSRGs on chromosomes. (**c**) The optimal lambda value was selected in the LASSO regression. (**d**) The LASSO coefficient profiles of the top 10 genes. (**e**) Relationship between the number of RF and error rates. (**f**) Ranking of the relative importance of genes in RF. (**g**) SVM-RFE algorithm for feature selection. (**h**) Venn diagram showing the feature genes shared by LASSO, RF, and SVM-RFE algorithms. ERSRGs, endoplasmic reticulum stress-related genes; EMS, endometriosis; PPI, Protein–protein interaction; LASSO, least absolute shrinkage and selection operator; RF, random forest; SVM-RFE, support vector machine-recursive feature elimination.

**Fig. 5:**
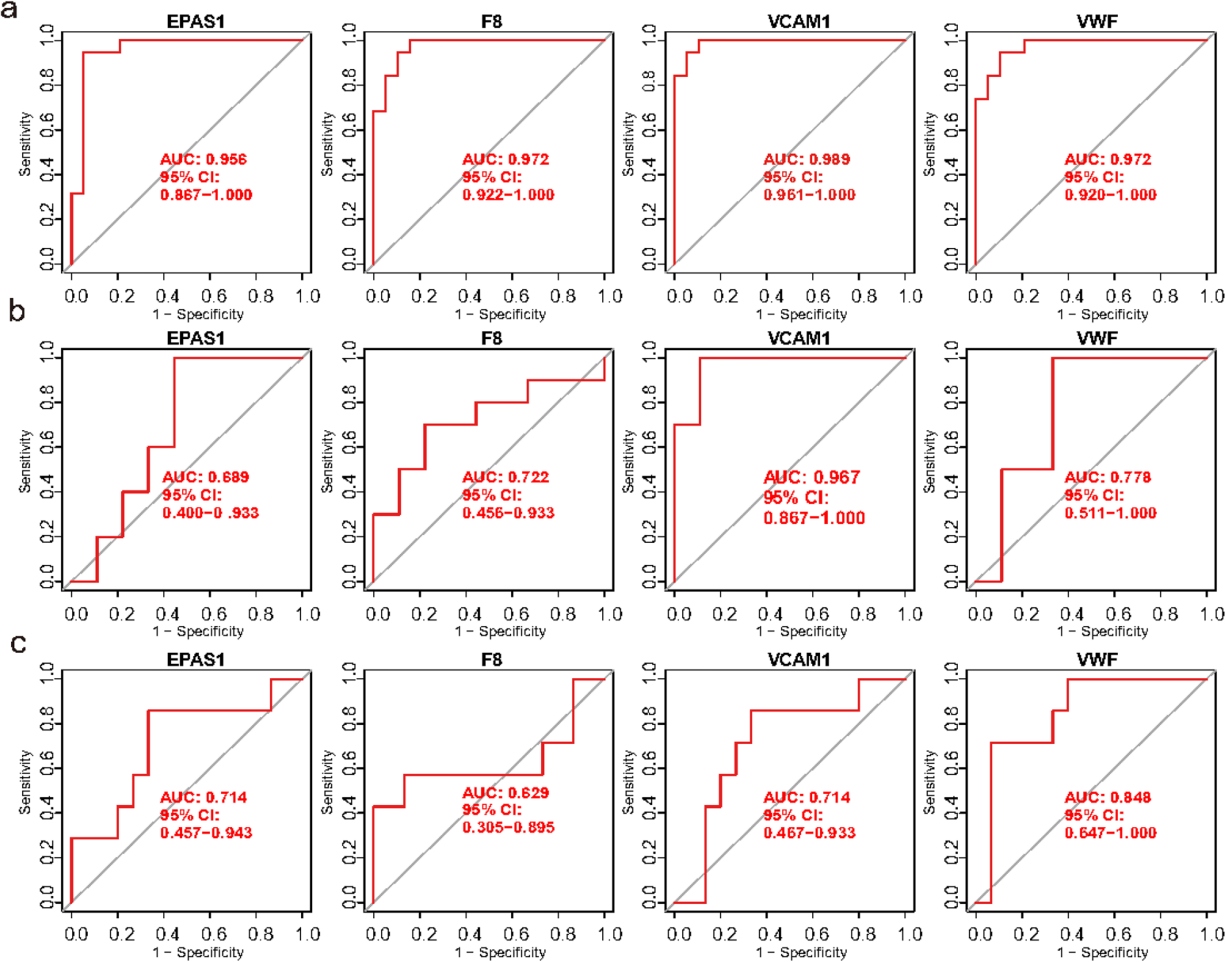
ROC curves were used to analyze the ability of four hub genes to distinguish between EMS and normal samples, and the AUC value was used to evaluate the distinction ability. (**a**) ROC curves in training set. (**b**,**c**) ROC curves in test sets GSE23339 (**b**) and GSE25628 (**c**). ROC, receiver operating characteristic curve; AUC, area under curve; EMS, endometriosis.

In this study, we combine multiple algorithms to construct a diagnostic model. PPI network analysis was performed on 29 genes. The cytoHubba of Cytoscape was used to determine the hub genes in the PPI network (Fig. 4a). Top 10 genes in network ranked by MCC method were obtained, namely ESR1, KPNA2, CAV1, TXN, CDK1, VWF, EZH2, VCAM1, EPAS1, F8. Figure 4b displays the chromosomal locations of top 10 ERSRGs that are differentially expressed. By looking at the location of gene family members on a chromosome. It is possible to determine whether they are distributed in clusters on the chromosomes. LASSO regression was performed on top 10 ERSRGs for feature selection and dimensionality reduction to exclude unimportant regulators, which ultimately identified 4 hub ERSRGs (Fig. 4c,d). For the random forest algorithm, six characteristic genes with relative importance > 2 were determined, including VCAM1, EPAS1, F8, VWF, ESR1, CAV1. (Fig. 4e,f). For the SVM-RFE algorithm, when the feature number was six, the classifier had the minimum error, containing VCAM1, EPAS1, TXN, F8, VWF, ESR1 (Fig. 4g). Following intersection, four characteristic genes shared by LASSO, RF, and SVM-RFE algorithms were finally identified (VWF, VCAM1, EPAS1, F8). (Fig. 4h) We calculate the average AUC of training set (Fig. 5a) and test sets (Fig. 5b,c), the average AUC values for all four genes exceeded 0.75 (EPAS1 = 0.786, F8 = 0.774, VCAM1 = 0.890, VWF = 0.866), demonstrating the good discriminatory efficacy of screened hub ERSRGs.

### Identification of ERS clusters

**Fig. 6:**
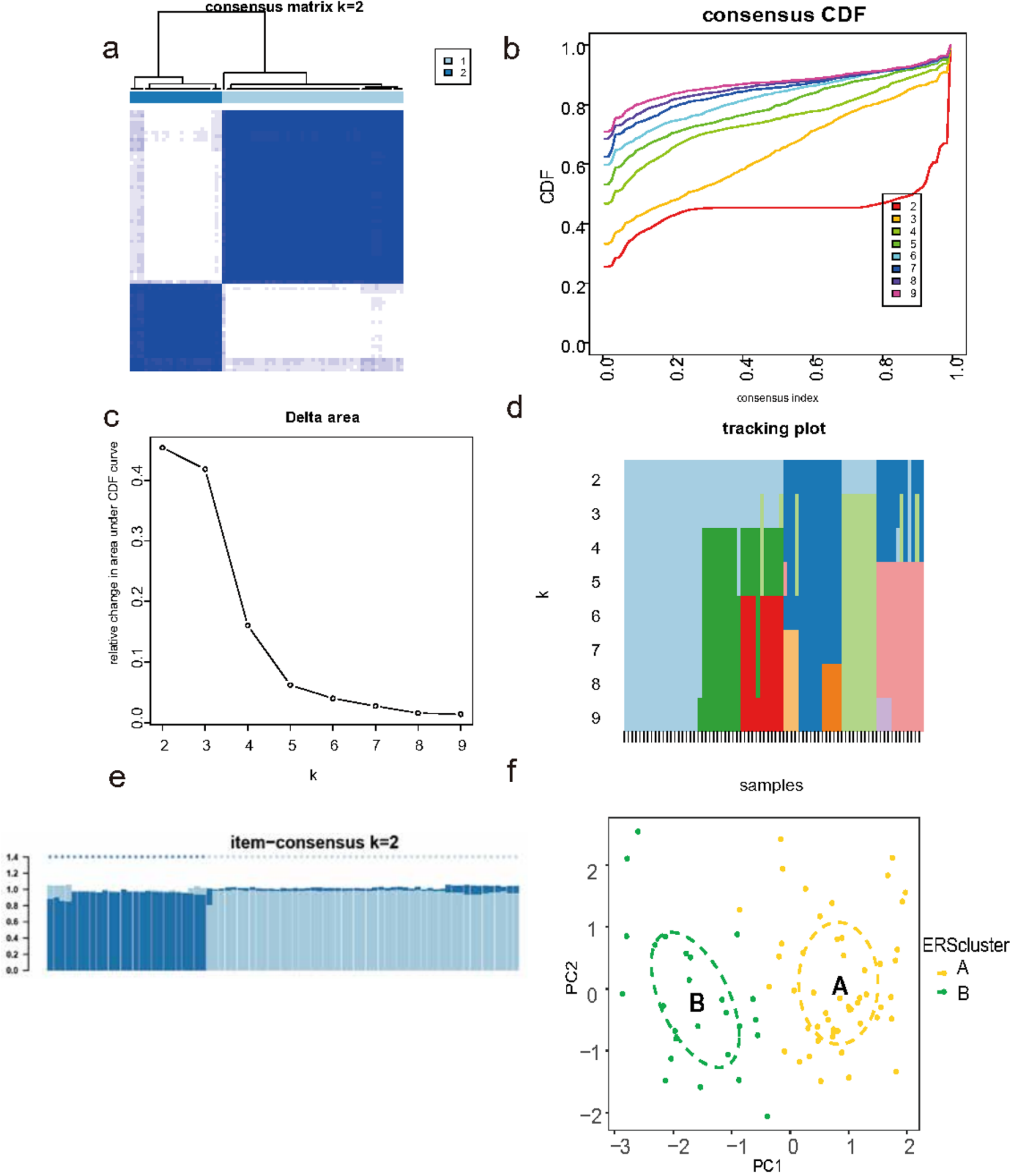
Identification two different ERS clusters in EMS. (**a**) Consensus clustering matrix when k = 2. (**b**) Cumulative distribution function (CDF) curves of clustering. (**c**) CDF delta area curves. (**d**) The tracking plot shows the cluster assignment of items (columns) by color for each k (rows). (**e**) Consensus clustering score of each cluster. (**f**) PCA visualizes the distribution of two clusters. ERS, endoplasmic reticulum stress; EMS, endometriosis; PCA, principal components analysis.

Four hub ERSRGs in EMS, including five up-regulated and five down-regulated genes, were used to cluster the EMS datasets GSE51981, which includes 77 endometriosis samples. We performed unsupervised consensus cluster analysis based on the expression of four hub ERSRGs using the “ConsensusClusterPlus” R package. We observed stable isoform numbers when k = 2, and significant differences in the relative changes in the area under the CDF curve from k = 2 to k = 9 (Fig. 6a–d). The consistency scores of the subtypes were all over 0.9 when k = 2 (Fig. 6e). We found two distinct ERS modification subclusters, with 51 samples in cluster A and 26 samples in cluster B.PCA verified the remarkable difference between the clusters (Fig. 6f).

**Fig. 7:**
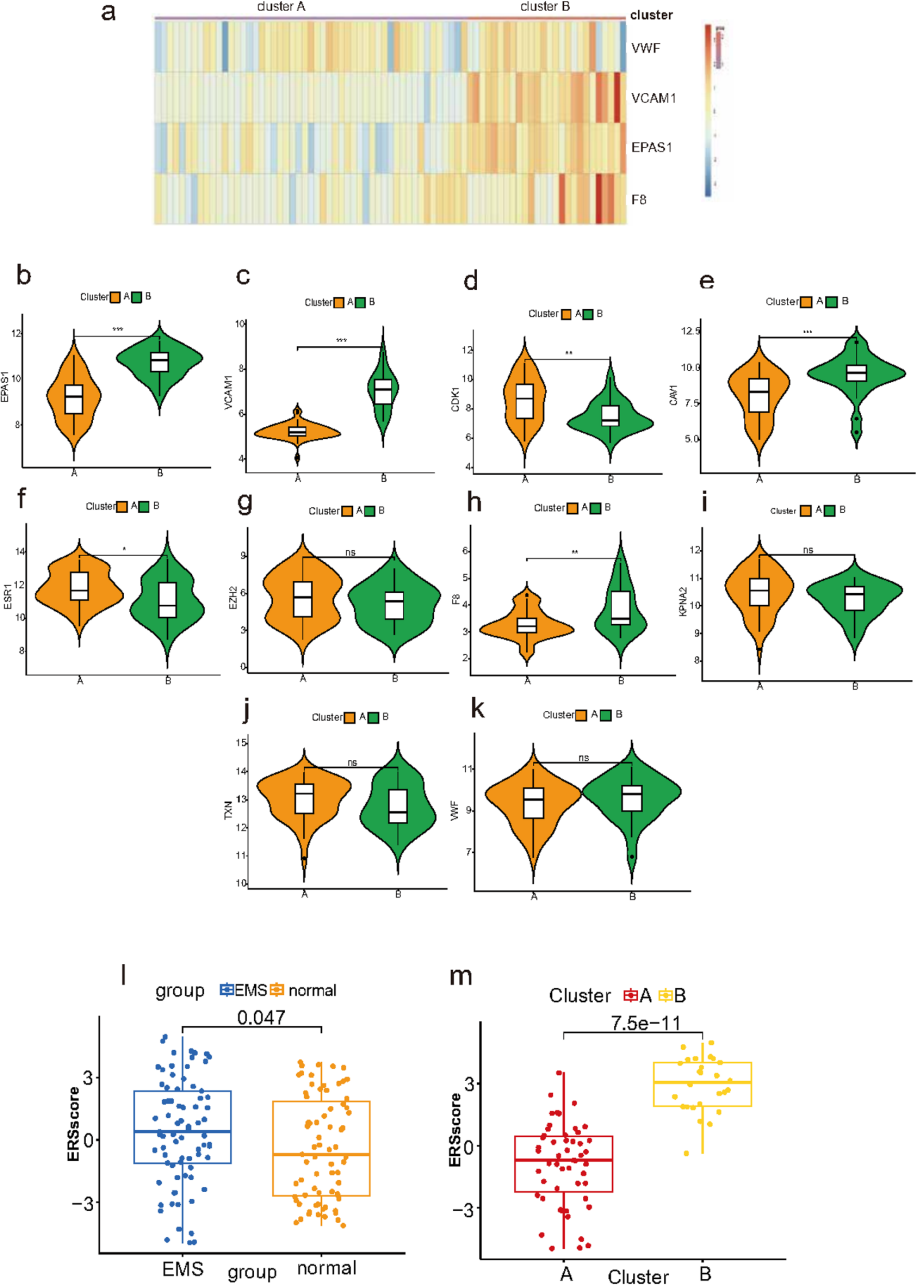
Hub gene expression and immune microenvironmental features between two ERS clusters. (**a**) Visualization of the expression of the four hub ERSRGs in cluster A and B. (**b**–**k**) Ten violin plots revealed significant differences in gene expression patterns between the two clusters. (**l**,**m**) Comparison of ERS score between EMS and normal samples (**l**), ERS cluster A and B (**m**). ERSRG, endoplasmic reticulum stress-related genes; EMS, endometriosis.

To better understand the molecular characteristics between subtypes, we evaluated the differences in the expression of 4 ERSRGs (Fig. 7a). The results showed that EPAS1, F8, VCAM1 presented a higher expression in B than A cluster. We used ten violin plots revealed significant differences in gene expression patterns between the two clusters (Fig. 7b–k), indicating that the various ERS clusters may have various transcriptome or other characteristics. We also calculated the ERS scores based on the four hub ERSRGs obtained above and compared them using the PCA method. The ERS score of EMS were higher than control samples (Fig. 7l) and ERS score of cluster B were higher than those of cluster A (Fig. 7m).

### Characteristics of the immune microenvironment in distinct ERS cluster

**Fig. 8:**
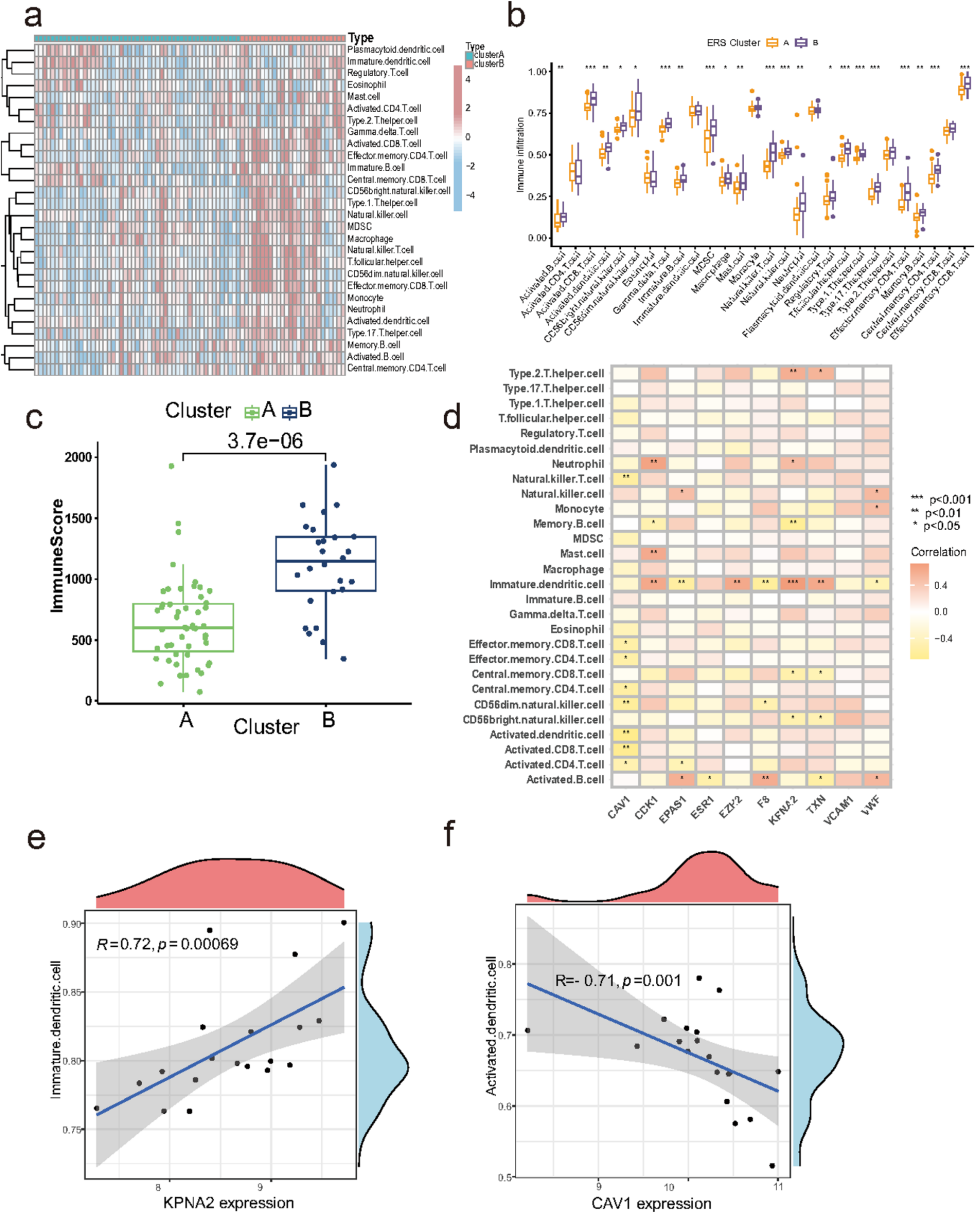
The abundance difference of infiltrating immunocytes between each cluster and gene-immune cell correlation analysis. (**a**,**b**) The heatmap (**a**) and boxplot (**b**) shows the immune cell abundance in two ERS cluster. (**c**) Comparison the immune score between the two cluster. (**d**) Gene-immune cell correlation heatmap. (**e**,**f**) The dot plot shows the most positively (**e**) and negatively (**f**) associated immune cells and genes. ERS, endoplasmic reticulum stress.

The concept of immune microenvironment was first proposed in tumors. Tumor tissues are composed of tumor cells, their surrounding immune and inflammatory cells, tumor-associated fibroblasts, and nearby mesenchymal tissues, microvessels, as well as a variety of cytokines and chemokines, which is a complex integrated system. Based on the available transcriptome sequencing data, it is clear that multiple immune cells infiltrate in endometriosis, a non-tumorigenic disease, and we therefore refer to and extend the concept of “immune microenvironment” to describe the infiltration of immune cells in endometriosis lesions. To identify differences in immune microenvironmental characteristics, we evaluated immune cells between these different ERS cluster. Significantly, Cluster B is clearly more immunologically active, while cluster A shows a relative defective immune cell infiltration. In Fig. 8a, we noticed that there was remarkable heterogeneity in in the abundance of immune cell infiltration between distinct cluster. Cluster B presented higher infiltration levels of activated B cell, activated CD8 T cell, activated dendritic cell, CD56bright natural killer cell, CD56dim natural killer cell, gamma delta T cell, immature B cell, MDSC, macrophage, mast cell, natural killer T cell, natural killer cell, neutrophil, regulatory T cell, T follicular helper cell, type1 T helper cell, type17 T helper cell, effector memory CD4 T cell, memory B cell, central memory CD4 T cell, effector memory CD8 T cell (Fig. 8b). After that, we calculated the immune scores of the samples using the ESTIMATE algorithm, the immune scores of cluster B were significantly higher than that of cluster A (Fig. 8c). Collectively, we identified cluster B as an immune subtype and cluster A as a less-immune subtype.

Furthermore, we explored the correlation between 10 ERSRGs and immune cells (Fig. 8d). Correlation analysis revealed that 10 ERSRGs were closely associated with most immune cells. For example, KPNA2 had the strongest positive correlation with immature.dendritic.cell abundance (r = 0.72) (Fig. 8e), and CAV1 had the strongest negative correlation with activated.dendritic.cell abundance (r = -0.71) (Fig. 8f). This indicates that KPNA2 and CAV1 play important roles in immunoinflammatory response in EMS.

### Explore the difference biological behaviors between ERS clusters

**Fig. 9:**
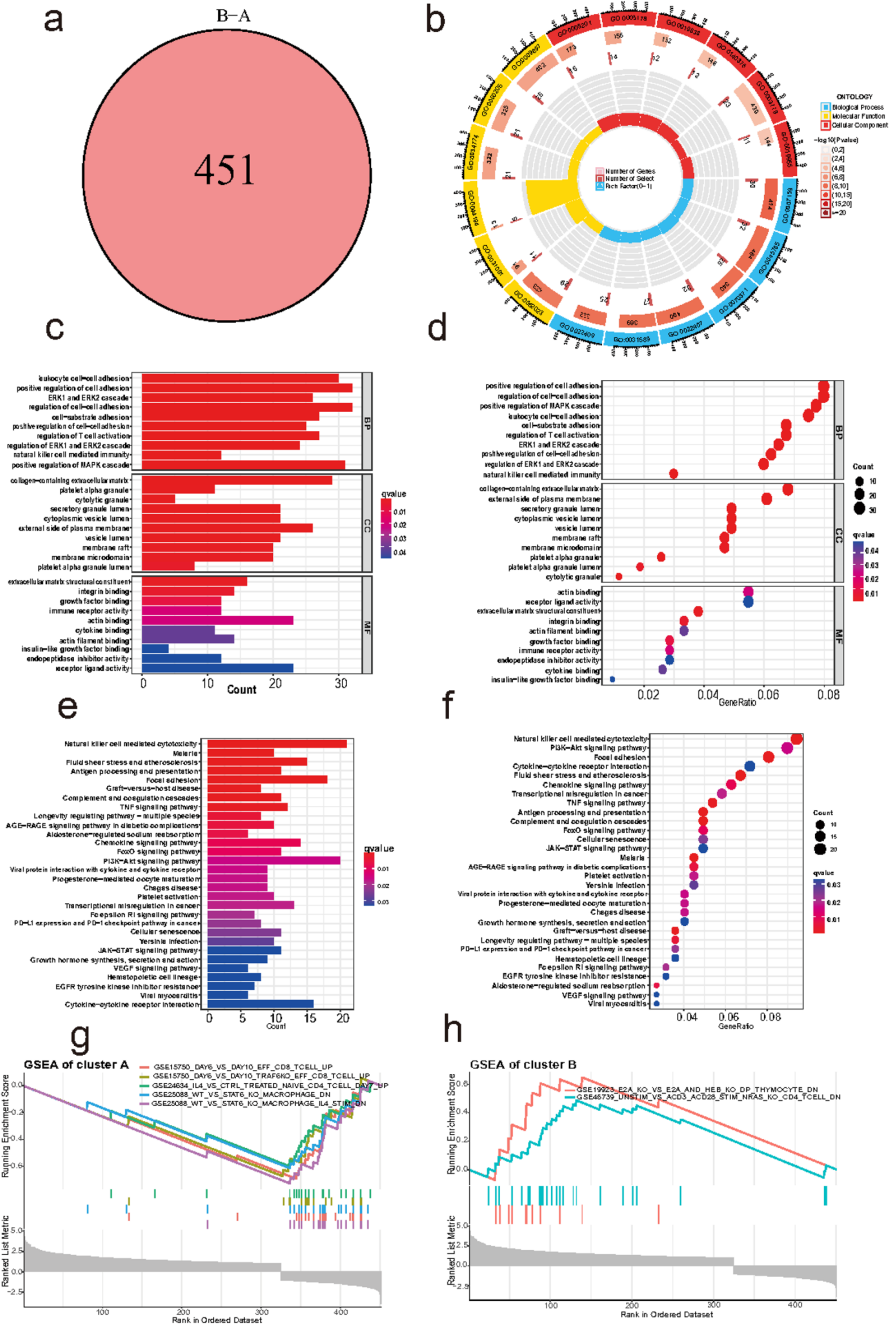
Functional analysis of genes involved in the ERS clusters in EMS. (**a**) 451 genes were related to the ERS clusters. (**b**–**d**) GO analysis showed the biological features of cluster DEGs. (**e**,**f**) KEGG analysis showed pathway enrichment of cluster DEGs. (**g**,**h**) GSEA enrichment analysis showing significantly activated immune-related functions in two clusters. ERS, endoplasmic reticulum stress; EMS, endometriosis; DEGs, differentially expressed genes; GO, Gene Ontology; KEGG, Kyoto Encyclopedia of Genes and Genomes; GSEA, gene set enrichment analysis.

We identified ERS cluster DEGs in order to explain the gene profiles related to cluster-mediated biological function regulation. A total of 451 cluster DEGs were screened out (Fig. 9a), including 127 DEGs with upregulation in cluster A and 324 DEGs with upregulation in cluster B. In the BP analysis of GO, cluster DEGs mainly participated in cell adhesion, regulation of T cell activation, ERK1 and ERK2 cascade, natural killer cell mediated immunity. In CC analysis, cluster DEGs mainly focused on collagen − containing extracellular matrix, external side of plasma membrane, vesicle lumen, external side of plasma membrane, cytoplasmic vesicle lumen and secretory granule lumen. MF analysis showed that cluster DEGs mainly related to extracellular matrix structural constituent, immune receptor activity, cytokine binding (Fig. 9b–d). In addition, in the KEGG analysis, the selected biological process of DEG enrichment significantly participated in biological pathways such as natural killer cell mediated cytotoxicity, PI3K − Akt signaling pathway, progesterone-mediated oocyte maturation, focal adhesion, chemokine signaling pathway, cytokine–cytokine receptor interaction pathway, VEGF signaling pathway, TNF signaling pathway and growth hormone regulation (Fig. 9e,f). We also conducted the GSEA analysis, the results showed that role of mammalian E proteins E2A and HEB in the development of T cells and N-ras in T cell development and function were significantly enriched in ERS cluster B (Fig. 9g), while functions TRAF6 regulated CD8 T cell memory development following infection by modulating fatty acid metabolism, extrathymic Treg development and STAT6 down-regulated in bone marrow-derived macrophages were significantly enriched in cluster A (Fig. 9h).

### Find potential drugs and clinicopathological features of ERS clusters

**Fig. 10:**
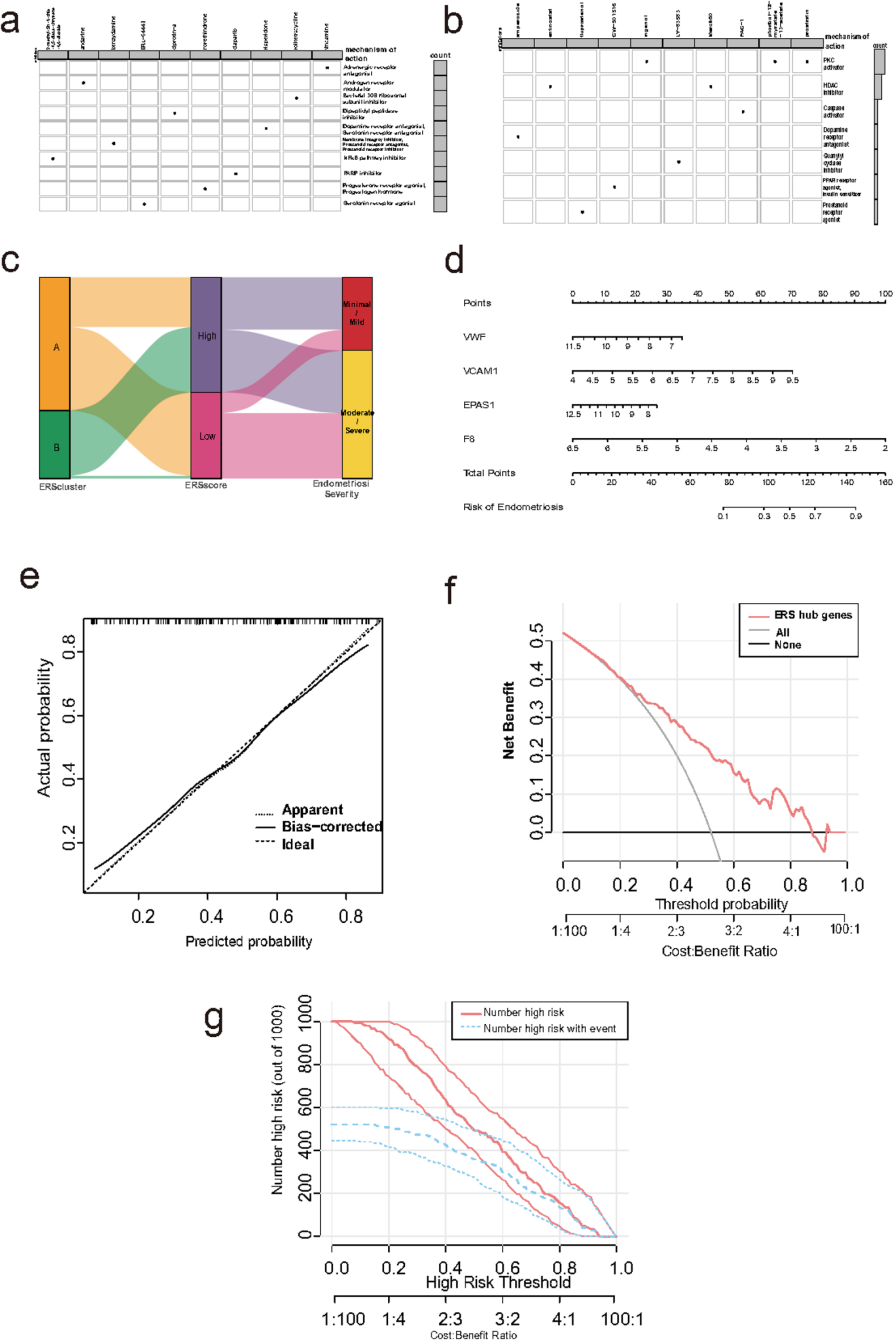
Therapeutic drug prediction, clinical characterization, and diagnostic modeling. (**a**,**b**) Heatmap displaying the mechanisms of action shared by prospective therapeutic drugs for cluster A (**a**) and cluster B (**b**), respectively. (**c**) Sankey diagram for two ERS clusters and clinical traits, ERS high and low score groups. (**d**) Representative nomogram for predicting the risk of EMS based on the four hub ERSRGs. (**e**) Calibration curves for evaluating the predictive ability of the nomogram model. (**f**) DCA curves for assessing the clinical value of the nomogram model. (**g**) Clinical impact curve of the nomogram model. ERS, endoplasmic reticulum stress; DCA, decision curve analysis.

We employed CMap database to identify potential therapeutic drugs for EMS. Differences between cluster A and cluster B were also apparent in compound prediction. Ten relevant compounds associated with distinct ERS cluster were identified. Next, we further evaluated the mechanism of actions (MOA) and drug target of these drugs to explore their potential mechanism for treating EMS. Notably, in cluster A, some adrenergic receptor antagonists, progesterone or progesterone receptor agonists, androgen receptor modulators, NF-κB pathway inhibitors, dipeptidyl peptidase inhibitors, and 5-hydroxytryptamine receptor agonists may have potential therapeutic roles (Fig. 10a), whereas in cluster B, some histone deacetylase inhibitors, protein kinase C (PKC) activators, PPAR receptor agonists and insulin sensitizers, adenylate cyclase activators, and caspase activators show a possible therapeutic role (Fig. 10b).

Sankey diagram to visualize the relationships between the ERS phenotypes and EMS severity. The Sankey diagram verified the cluster A was associated with low ERS score, moderate or severe EMS. However, in cluster B with high ERS scores, mild EMS and moderately severe EMS had distributions with little difference. It is evident that the degree of clinical manifestation of EMS has greater heterogeneity among different ERS clusters and even within ERS cluster (Fig. 10c).

We constructed a nomogram (Fig. 10d) to evaluate its predictive power using the calibration curve to predict the risk of EMS more clearly (Fig. 10e). Decision curve analysis (DCA) (Fig. 10f) and clinical impact curve (Fig. 10g) indicated that the “nomogram” curve was higher than the gray line. The calibration curve indicated a minimal difference between the real and predicted EMS risks, suggesting that the nomograph model of EMS is precise.

### Validation mRNA and protein expression in collected human endometrial tissue

**Fig. 11:**
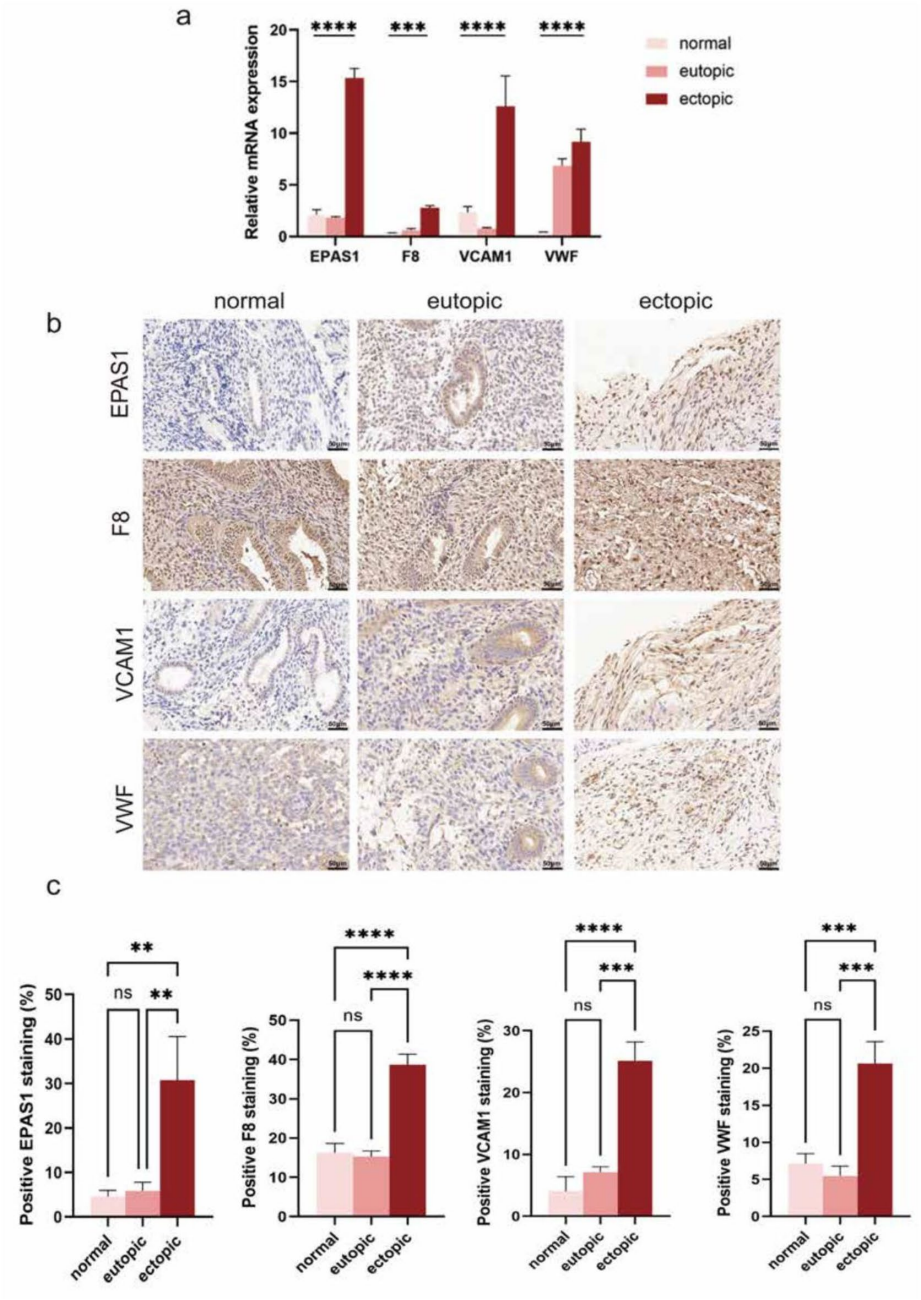
qRT-PCR and IHC validation the hub gene expression level. (**a**) Hub ERS genes mRNA expression of normal, eutopic and ectopic endometrium. (**b**) Immunostaining of EPAS1, F8, VCAM1, VWF protein expression. (**c**) Semi-quantitative analysis of IHC results. qRT-PCR, real-time quantitative reverse transcription; IHC, immunohistochemistry. *p < 0.05, **p < 0.01, ∗  ∗  ∗ *p* < 0.001, ****p < 0.0001.

The results of bioinformatics analysis suggested that EPAS1, F8, VCAM1, and VWF might be highly expressed in endometriosis. After RT-qPCR, the mRNAs of the four ERS hub genes in ectopic endometrium were significantly higher than those in the eutopic endometrium and normal control endometrium (Fig. 11a). We performed immunohistochemical staining on normal control endometrial specimens, ectopic endometrial specimens and eutopic endometrial specimens in order to detect the protein expression. The results showed that the protein expression levels of EPAS1, F8, VCAM1, and VWF were consistent with those of mRNA (Fig. 11b,c).

## Discussion

Currently, there is a lack of biomarkers with both accuracy and sensitivity for the diagnosis of endometriosis. Endometriosis is an estrogen-dependent inflammatory immune disorder. In addition to surgical removal of endometriosis lesions and loosening of pelvic adhesions, hormonal therapy remains the first line of pharmacologic treatment for endometriosis. Therefore, it is necessary to find the hub genes associated with the diagnosis of endometriosis and to explore the role of these hub genes in the pathogenesis of endometriosis, and drug therapy. In this study, we explored the role of endoplasmic reticulum stress-related genes in endometriosis immune infiltration, disease typing, potential therapeutic agents, biological functions, and pathways.

Our study confirmed that the levels of infiltration of a wide range of immune cells were significantly elevated in ectopic lesion samples, which is consistent with previous research, suggesting the presence of a heavy immune-inflammatory response in endometriosis. Several studies in recent years have mechanistically confirmed the relationship between endoplasmic reticulum stress and inflammatory immune responses. For example, endoplasmic reticulum stress-induced inflammation and production of the pro-inflammatory cytokine IL-6 have been reported to be dependent on two members of the NOD-like receptor family, NOD1 and NOD2^18^. Another study on inflammatory bowel disease confirmed that endoplasmic reticulum stress induced by deletion of the transcription factor X-box binding protein-1 (XBP1) can lead to aberrant responses of intestinal epithelial cells to inflammatory signals^32^. In a study of endometriosis, treated endometrial stromal cells with peritoneal fluid from patients with endometriosis and normal control patients, the result suggested that endoplasmic reticulum stress-associated UPR pathways were activated in endometriosis^33^. Our study also showed that a relatively high degree of endoplasmic reticulum stress was present in ectopic lesion samples of endometriosis and that different endoplasmic reticulum stress subtypes had different degrees of UPR activation. However, initial activation of the UPR contributes to the endoplasmic reticulum’s emergency response to adverse external signals, and over-activation leads to cell death, which is the entry point for many current studies in related fields to confirm the therapeutic effects of drugs on endometriosis. Our study of endoplasmic reticulum subtypes used the GSE51981 database, and Sankey plots showed that the degree of endoplasmic reticulum stress did not significantly correlate with the degree of clinical manifestations of endometriosis in this dataset. Is the specific level of endoplasmic reticulum stress in endometriosis associated with case typing and disease severity? This question remains to be further elucidated using large-scale transcriptomic data and clinical samples.

Estrogen and progesterone strictly regulate the physiologic cycle of the endometrium. These steroids also have receptors in the endoplasmic reticulum that are involved in the regulation of protein folding, calcium homeostasis in the endoplasmic reticulum, and degradation of misfolded proteins. Abnormal endoplasmic reticulum stress response to progesterone enhances the invasiveness of endometrial stromal cells in endometriosis through the AKT/mTOR pathway, which has attracted our interest in the role of endoplasmic reticulum stress in endometriosis^2^. In this article, for the first time, we found the hub endoplasmic reticulum stress-related genes in endometriosis by bioinformatics methods and performed endoplasmic reticulum stress typing in endometriosis. First, we identified 10 characterized endoplasmic reticulum stress genes by differential analysis, WGCNA, PPI and Cytoscape. Second, machine learning was used to further screen these 10 genes for endoplasmic reticulum stress-centered genes. Subsequently, unsupervised clustering was performed using the screened VWF, VCAM1, EPAS1 and F8. We categorized endometriosis into two subtypes, A and B, which showed significant differences in the degree of immune cell infiltration and the type of immune cell infiltration. type B had a higher endoplasmic reticulum stress score and tended to be an immune cell-rich type, whereas type A had a lower endoplasmic reticulum stress score and a relatively low immune cell infiltration. In the comparative analysis of fractional immune infiltration, we found that several immune cell types with *p* < 0.001, such as activated B cells, CD56 strongly positively expressing NK cells, immature B cells, myeloid-derived suppressor cells (MDSC), NKT cells, NK cells, regulatory T cells, follicular helper T cells, and helper T cells 1 (Th1), which have also been shown to be associated with immunomodulation in endometriosis. For example, in a 1995 study, researchers found a strong correlation between NKT cell-mediated lysis of ectopic endometrial cells and downregulation of HLA1-like receptors^34^. Patients with severe endometriosis had significantly higher levels of CTLA-4^+^T cells than those with mild endometriosis. In addition, CTLA-4^+^T lymphocytes were negatively correlated with the percentage of NK and NKT-like cells in women with both endometriosis and infertility^35^, which indicate that immunologic mechanisms of associated infertility may differ between endoplasmic reticulum stress subtypes and endometriosis clinicopathologic types and degrees of clinical manifestations of endometriosis.

In the functional enrichment analysis of genes differing in endoplasmic reticulum stress subtypes, we noted significant functional enrichment of genes for extracellular matrix and cell adhesion. Endoplasmic reticulum stress enhances leukocyte recruitment through extracellular signals, remodels the immune microenvironment, and alters the behavior of immune cells through the secretion of polarizing cytokines, and in doing so restores tissue protein homeostasis. Muscle cells and airway epithelial cells have been reported to secrete a functional leukocyte-adherent hyaluronic acid matrix after various forms of endoplasmic reticulum stress^36^. Endometriosis lesions are highly resistant to apoptosis and cell adhesion, and attenuating endoplasmic reticulum stress-induced ectopic cell adhesion may be an important treatment for endometriosis. A related study showed that Frankincense could alleviate endometriosis by reducing the adhesion and proliferation of ectopic endometrial cells through endoplasmic reticulum stress/p53-apoptosis and chemokine-migration/adhesion pathways^37^. Notably, activated T cells showed significant enrichment in functional enrichment analysis and gene-immunocyte correlation analysis. We found that CAV1 was negatively correlated with multiple T cell subtypes. In a previous study, knockdown of Sirt1 in endothelial cells induced endoplasmic reticulum stress and miR-204 expression in endothelial cells, decreased CAV1, and impaired endothelium-dependent vasodilation, but there are no studies on CAV1 in relation to endometriosis^38^. In addition, we found a positive correlation between immature dendritic cells (iDC) and several endoplasmic reticulum-related genes. The proportion of iDCs was increased in the peritoneal cavity of patients with endometriosis compared to mature dendritic cells (mDCs), and maturation of dendritic cells in the peritoneal cavity plays an important role in the development of endometriosis^39,40^. However, in our immune cell differential analysis, there were no significant differences in iDCs between endometriosis and control samples, or in endoplasmic reticulum stress cluster A versus cluster B.

Our study identified four endoplasmic reticulum stress center genes, VWF, VCAM1, EPAS1, and F8. VWF is a macromolecular plasma protein that plays a key role in maintaining normal coagulation and contributes to thrombotic disorders following endothelial and platelet dysfunction. The VWF gene is expressed predominantly in vascular endothelium and megakaryocytes, and its expression level is commonly used as a measure of angiogenesis capacity^41,42^. A Mendelian randomization study based on GWAS data from a large population showed a causal relationship between elevated VWF and an increased risk of endometriosis^43^. In lesions treated with GnRH agonists (GnRH-a), microvessel density was significantly reduced in patients with positive VWF expression^44^. However, protein expression of VWF did not show r-ASRM stage-dependent changes in endometriosis^45^. VCAM1 is a cell adhesion molecule, which is often used as a relevant measure of inflammation and malignancy cell adhesion capacity in studies related to a number of diseases, and has also been associated with transvascular endothelial migration of immune cells^46^. And estrogen-mediated upregulation of VCAM1 contributes to mast cell recruitment and differentiation^47^. As a classical inflammation-associated gene, knockdown of VCAM1 impedes TGF-β1-mediated endometrial cell proliferation, migration and invasion as well as attenuates inflammatory responses in endometriotic lesions^48,49^. In 2017, researchers confirmed that VCAM-1 levels were higher in both ectopic endometrial tissue than in the native endometrium. And mRNA levels of VCAM-1 were higher in normal peritoneal tissue samples from women with endometriosis compared to control normal peritoneal tissue samples. In terms of serum protein levels, VCAM-1 levels were higher compared to controls^50^. Subsequently another researcher in 2020 explored whether urinary VCAM1 protein expression levels could be used to predict endometriosis, but the results confirmed that there was no significant difference in urinary VCAM1 protein levels between patients and non-patients. Our study was based on transcriptome sequencing of endometrial tissues, and in follow-up experiments, we verified that the protein and mRNA expression levels of VCAM1 in ectopic endometrial tissues were higher than those in normal control endometrium. This suggests to us that serum combined with VCAM1 testing of tissues might be a new tool for diagnosis or preoperative prediction of potential patients with endometriosis^51^. Mechanistic studies of endometriosis associated with EPAS1 are lacking, but it is certain that EPAS1, as a key transcription factor in cellular response to hypoxia, is closely associated with inflammatory response and angiogenesis under hypoxic conditions, and more importantly, EPAS1 expressed in the endometrial stroma has been associated with invasion of trophoblast cells during embryo implantation, and mice knocked out of EPAS1 expression were infertile due to infertility due to failure of implantation^52,53^. Whether EPAS1 is involved in endometriosis-associated infertility may be a new research direction for the future. A study in 2018 confirmed that EPAS1 expression was significantly upregulated in CD73^+^CD90^+^CD105^+^ pluripotent stem cells isolated from ectopic endometrium compared to paired in situ endometrium, which may be associated with the progression of ovarian endometriosis to associated ovarian cancer^54^. The F8 gene is required for the production of coagulation factor VIII, which is essential for clot formation. In a mouse endometriosis model study, researchers found that the F8 antibody had a strong targeting effect on endometriotic tissues, based on which the study significantly reduced the size of endometriosis lesions using the F8 antibody-IL-10 fusion protein^55^, and a similar study found that the F8 antibody-IL4 can similarly reduce the size of endometriosis lesions^56^. We look forward to the future discovery of upstream and downstream pathways and other molecules interacting with these four key genes, and to the thorough elucidation of the involvement of endoplasmic reticulum stress-related mechanisms in the pathogenesis and potential therapeutic drug molecular biology of endometriosis.

There exists significant heterogeneity in clinical symptomatology and disease severity among individuals with endometriosis; individuals exhibiting high ASRM and EFI scores may manifest mild or asymptomatic pain, while those presenting with minimal lesions or mild pelvic adhesions observed laparoscopically may experience more severe pain. Within the scope of our investigation, the classification and grading of endoplasmic reticulum stress did not demonstrate a significant correlation with the severity of endometriosis. There is a notable absence of diagnostic markers demonstrating high sensitivity and specificity for endometriosis. The diagnostic model constructed in this investigation underwent validation through diverse algorithms, demonstrating favorable performance in discriminating endometriosis samples from control endometrial specimens. Furthermore, the endoplasmic reticulum stress-associated core genes identified herein hold promise for clinical application in the diagnosis of endometriosis.

Presently, the primary modalities for addressing endometriosis encompass laparoscopic surgery and hormonal therapy. Moreover, our investigation has delineated compounds that target distinct subtypes of endoplasmic reticulum stress, potentially offering novel avenues for both scientific inquiry and clinical intervention in the management of endometriosis. It is reasonable to hypothesize that these compounds may promote the development and progression of endometriosis by inducing or inhibiting endoplasmic reticulum stress. Interestingly, our study found that subtypes with high endoplasmic reticulum stress scores also had higher immune cell infiltration scores, and this feature was not only reflected among endoplasmic reticulum stress subtypes, but also showed the same trend between endometriosis samples and control samples.

Our study also presents certain limitations. Specifically, we did not investigate the potential association between endoplasmic reticulum stress subtypes and clinical phenotypes of endometriosis. It is pertinent to inquire whether disparities exist in endoplasmic reticulum stress-related gene expression profiles and the associated immune cell infiltration patterns between ovarian and non-ovarian endometriosis. Additionally, despite the utilization of multiple algorithms to validate the accuracy of our diagnostic models, the diagnostic utility of endoplasmic reticulum stress-related genes in endometriosis diagnosis warrants further substantiation through comprehensive investigations. Currently, a variety of machine learning algorithms are used to build diagnostic models. We will continue to learn new methods and combine multiple algorithms in the subsequent research to establish more accurate diagnostic models, hoping to provide suggestions for clinical endometriosis diagnosis. Moreover, the absence of publicly available transcriptome sequencing data encompassing samples representing diverse pathologic types of endometriosis poses a challenge, and the outcomes of bioinformatics analyses may be subject to certain biases attributable to variations in sequencing platforms, statistical methodologies, and databases employed. In addition, this study only analyzed RNA transcriptome data at the tissue level, without involving single-cell transcriptomics or spatial transcriptomics. Currently, several single-cell transcriptomic studies have revealed the characteristics of epithelial and stromal cells in endometriosis, and significant differences in single-cell transcriptomics exist among different pathological types of endometriosis. Notably, both epithelial cells and stromal cells in ovarian endometriosis show dysregulation of pro-inflammatory pathways and upregulation of complement proteins. Single-cell transcriptomics and spatial transcriptomics offer new dimensions for endometriosis research. The former reveals the heterogeneity of ectopic endometrial cells and abnormal expression of key subpopulations, while the latter deciphers the spatial arrangement of cells and their interactions with the microenvironment. Together, they provide high-precision research tools and new directions for elucidating the molecular mechanisms of lesion formation and developing precise targeted therapies. Finally, this study did not use cell lines ore animal models to validate the screened genes, immune cells, and potential therapeutic compounds. In order to draw more convincing conclusions, further studies on the involvement of endoplasmic reticulum stress-related genes in the pathogenesis of endometriosis are necessary next.

## Conclusions

The expression profiles of key endoplasmic reticulum stress-related genes, including EPAS1, F8, VCAM1, and VWF, exhibited significant disparities between endometriosis specimens and both adjacent normal endometrial tissues and control endometrial tissues. These pivotal genes were utilized for stratifying endometriosis cases into distinct immune and non-immune subgroups. Furthermore, distinct patterns were observed in the expression levels of endoplasmic reticulum stress-associated genes, endoplasmic reticulum stress scores, immune cell infiltration levels, biological functionalities, signaling pathways, and potential therapeutic targets across different endoplasmic reticulum stress subtypes of endometriosis. Notably, a diagnostic model based on the expression profiles of endoplasmic reticulum stress-related genes demonstrated robust discriminatory capacity for identifying endometriosis cases.

## Supplementary Information

Supplementary Information 1.

Supplementary Information 2.

## Data Availability

The public datasets GSE7305, GSE11691, GSE23339, GSE25628 and GSE51981 used in this paper are available on the NCBI website (https://www.ncbi.nlm.nih.gov/geo/).
