# Supplementary material for: Identified endoplasmic reticulum stress-related molecular cluster and immune characterization in endometriosis
Source: Sci Rep. 2025 Nov 4;15:38553. doi: 10.1038/s41598-025-22400-9 (PMC12586515; doi:10.1038/s41598-025-22400-9)

Supplement Material 1

1. 1450 ERS related gene

ERN1 HSPA5 ATP2A2 ATP2A1 SERP1 ATP2A3 XBP1 EIF2AK3 ERP29 SERP2 ERP44 KDELR1 OS9 ERAP1 ERO1A KDELR2 HERPUD1 ERLEC1 VCP ERAP2 KDELR3 SEC16A ERN2 ATF6 ERP27 RER1 ERO1B DDIT3 CALR CHERP TP53 RYR2 HSP90B1 ERMP1 CANX EIF2S1 ATF4 RYR1 CPT2 TRDN SYVN1 PDIA3 CASQ2 NFE2L1 MAPK8 DERL2 NFE2L2 DERL1 LMAN1 DNAJC10 TXNDC12 CASQ1 HMOX1 APP SOD1 CALM1 P4HB PSEN1 HYOU1 MAPK14 CASP3 ITPR1 SIL1 CASP4 DNAJB9 AMFR PRKN CAT MAP3K5 MAPK1 BCAP31 BCL2 SEC23A DNAJC3 H6PD STIM1 TNF PDIA4 CLU G3BP1 CREB3 SLC6A4 TXNDC5 PPP1R15A JSRP1 DERL3 STIP1 BAX INS MAN1B1 MANF ATF6B LNPK OXSR1 RTN4 CRH EPM2A CFTR DNAJB11 WFS1 STING1 FKBP14 SEL1L CREB3L1 HSPA1A FOS PARK7 MTOR ATP13A1 HSPA4 SELENOS IL6 DDRGK1 MIA2 LMNA KCNQ1 KCNH2 HSF1 SIRT1 UBC ERGIC3 BDNF SEC61A1 SREBF1 SIGMAR1 G3BP2 RPN1 SELENON HSPA8 PDIA2 IL1B PTPN1 CYCS HSP90AA1 ERLIN2 BAG6 SURF4 VAPB CXCL8 INSIG1 GSR NHLRC1 NOS3 NR3C1 PKP2 SREBF2 HSPB1 CREB3L2 SELENOK APOE ANK2 DDX3X JUN SEC31A TMED4 SCAP SEC24A SOD2 MIA3 HMGCR TGFB1 SNCA ADIPOQ RTN3 TXN SCN5A EDEM1 EIF2AK2 ASPH NOTCH3 RPS27A LMAN2 BSCL2 CREB3L3 SEC13 RAB1A CKAP4 PSEN2 PARP1 TRAM1 PTGS2 ERLIN1 TAPBP MAPK10 RRBP1 SQSTM1 RAB1B ERGIC2 SEC24B CRHR1 ESR1 CASP9 CLN3 CALM3 DMD VWF ARL6IP1 TOR1A SERPINA1 CASP8 UFL1 STIM2 YIPF5 BNIP1 P4HTM ALG1 CRP ATF3 CAV3 ATL3 MPO SESN2 PPIB CISD2 LRRK2 TAP1 KEAP1 POMC TMED2 VEGFA AKT1 INSIG2 CD4 PLN UGGT1 HSPA9 JPH2 NOX4 EIF2AK1 ABL1 RSAD2 APOB SAR1B COPB1 SLN CCL2 HERPUD2 GJA1 SPAST SGK1 STX17 SEC62 CALM2 GET3 TRIM13 SEC24C HLA-B CACNA1C ORAI1 DDOST TMBIM6 PRNP PDIA6 EIF4G1 BCL2L1 PRKCD EGFR GBF1 SLC37A4 UBE2J1 PDCD6 SEC63 SEC61B EDEM2 ALB ESYT1 VIM CAPN3 TARDBP DNAH8 ATL1 MYOC MAPK9 XDH CANT1 RNF139 SERPINH1 HTRA2 PREB OSBPL8 CAV1 TAP2 TMED10 BAK1 PRDX4 TMEM33 TFG SOAT1 SAR1A HSD17B10 STUB1 EDEM3 FOXO1 PINK1 RYR3 RTN1 VHL PRKAA1 TEX264 VAPA SEC24D RPN2 CYBA TRIP11 BACE1 TOR1B FOXO3 TLR4 AUP1 RNF185 TECRL TMED9 GSK3B HSPA1B NFKB1 UBQLN1 RCN2 TRAF2 MOGS ATM DHCR24 PCSK9 NOS2 STX18 FMR1 PMM2 UGGT2 STX5 FAF2 AHCYL1 FKBP5 TMEM208 KCNE1 G6PC1 CEBPB MAPK3 UBA52 TNFRSF10B CCDC47 SLC8A1 TXNIP IER3IP1 CCDC88B KCNJ5 QRICH1 EGF CYP2E1 COMP FOXRED2 PIEZO1 COMT BBC3 MAPT NQO1 RAB10 G6PD RAB6A NLRP3 PON1 COL7A1 HLA-A STARD3 BECN1 CYP1A2 LOC110806262 EMC1 MAPKAPK2 COPA TG CDKN1A TOR1AIP2 SEC23IP ESYT2 AQP11 SRPRA DSP EIF2AK4 KTN1 FKRP POGLUT2 GPR37 PRKAA2 TMX3 GAPDH CYBB THBS1 DNM1L VKORC1 F2 SLC2A1 UBB HSPD1 TRPV4 CYB5R3 APEX1 BCL2L11 MZB1 SEC22B SHH MAP2K6 CRYAB ATL2 CYP1A1 SEC23B KCNE2 ZDHHC6 DHCR7 USO1 TMX2 DMPK SMPD1 SRP68 HTT SRC GPX1 UBE2G2 ITPR3 HIF1A MAP1LC3A CLN6 GABARAPL1 OXT OSBP MAPK13 GPER1 NOTCH1 P3H4 MDM2 NPC1 GBA DPAGT1 MAP2K7 UGT1A1 NUPR1 SCD PKD2 SCAPER ICMT HRC LDLR F9 BET1 TMX1 CACNA1S MBTPS1 AGR2 CNIH4 S100A1 JPH4 CASP7 PPARG PCSK6 BOK SMPD4 DRD2 UBXN8 UGT1A6 TMEM214 ALG13 SEC61G RHOA RNFT1 PITPNB PRDX2 STT3A ARL6IP5 EMC10 CREB1 AKAP9 PLEKHF2 VCAM1 PRDX1 VMP1 MAOA PIGN MTTP MAP1LC3B EPO BRSK2 JAK2 STAU1 MAP2K4 GABARAPL2 INPP5K PIK3R1 HM13 TMCC1 ZW10 DNAJB12 CASP12 RINT1 RNF186 TRIB3 SDHA FICD UBE2J2 RAB2A LONP1 BRCA1 SNTA1 HFE MUC1 HSP90AB1 IL10 PON2 SDF2L1 AIFM1 CAMK2G TMED7 EP300 PRDX6 OSBPL3 TUSC3 EDN1 JPH1 MICB ORMDL3 C9orf72 TMED1 ELN GOLGA2 TMTC3 SHISA5 TF EBP SLC39A14 PTEN CTNNB1 GSTM1 UBA5 HMGB1 IFNG NR3C2 SCP2 UBXN4 CERT1 ATG14 F8 GRIA1 GABARAP RCN1 HSD11B1 CARD14 AKAP6 RFT1 PIGK GH-LCR TSPO P3H1 POMT2 ATR RAC1 AGR3 ALG14 ADRB2 NCK1 PTGS1 MPPE1 SP1 TMCO1 YKT6 PPP1R15B EEF1A1 LBR ZC3H12A NAGLU CASP2 MBTPS2 CYP2D6 ITPR2 CDK1 CNR1 FKBP1B ATP1A3 INSR GRIN1 HACD2 GET4 MCFD2 IGF1 GRAMD1A CALHM1 DBH PIK3C3 SCFD1 SACM1L GLA ALG2 TLR9 UBL4A GET1 MYH7 MAPKAP1 PDZD8 CREBRF SHC1 ERMARD HMGCLL1 UBE2K KDR SELENOF HSPA13 CLN8 LPCAT3 KCNJ11 JKAMP DLD ZFAND2B BCL2L10 JPH3 EEF2 SEC11A GPX7 EMC7 HGSNAT PABPC1 KDSR SGTA SCAMP5 GANAB G6PC3 NDRG1 MOSPD2 UFM1 NR1H2 SOAT2 MAP2K1 GSTP1 PRKCA UNC93B1 YIF1A PTPN2 SGPP1 MSRB3 CDKN3 KIF1C UMOD MX1 LACC1 NSF MFN2 CHAT ACTB GORASP1 SCARA3 TFRC CDK5RAP3 REEP4 MGST1 GORASP2 PDHA1 PRL FKBP4 GRAMD1B AGER IL1A ANXA5 TECR PROC POR CHRNE NBAS SORT1 REEP1 CTSD SUMF2 HTR2A SRP54 FAS PRDX3 STARD3NL PTGIS TGM2 DGAT1 STAT3 SET KCNJ2 CCND1 PKD1 HSD17B12 ANK1 TIA1 NOTCH2 TMEM259 UBAC2 ZFYVE27 CDK5 HAX1 NOL3 MICA MR1 CP HLA-C PKM STT3B RETREG1 DPM1 RNF183 TRPA1 SRPRB DNAJB14 PPM1L IKBKG DES CTSB SSR2 NGLY1 CLCC1 COPE CREBBP PPP3CA NOS1 PML MTHFR CRHR2 ICAM1 MAN1A1 MAP2K3 ZFYVE1 RNF5 CYP1B1 PACS2 PIK3CG FURIN ECPAS ELAVL1 SYT2 ABCD1 EIF4E EMC3 TRPM2 PITPNM1 LEP CLGN SLC35B1 F5 KRAS RHBDD1 C1R TRAPPC11 POMT1 SSR1 DUSP19 GHRL NLRP1 TRAPPC2 CYP3A4 SRP72 ZMPSTE24 MYC PPARGC1A NPY CYP2C19 EMC4 GATA1 MGAT2 KPNB1 SEC31B DNAJB2 BTRC CD74 BRCA2 CLCN1 PRDX5 ADCYAP1 EIF2B5 FN1 CYB5A DSPP CD36 TANGO2 DST ATXN2 STK25 MYDGF PLOD3 TNFRSF1A KNG1 SCYL1 OXTR ABCD4 TMEM117 RTN2 CDC42 DNAJB1 BCAP29 EMC2 PLA2G6 S100A9 GBA2 CALU ACTC1 ACTA1 EMC6 GADD45A JAGN1 ATF2 MMGT1 FANCD2 TMEM43 COL4A1 CYB5R4 PLOD2 TERT SSR4 VCPIP1 SLC25A1 LMBRD1 USP19 TRAPPC3 C1S NOS1AP GOSR1 HLA-DRA ELOVL4 SRP14 VAMP7 NPLOC4 SAMD8 RNF13 SVIP REEP5 YWHAE MARCHF6 POP1 TTN TTR TYR SGPP2 TAPBPL KCNA2 CIRBP SLC39A7 FKBP10 EMC8 GRIN2A ACSL4 HNRNPK CPQ COL2A1 ADCYAP1R1 RAB18 SFTPC PIGT SRI CES1 FKBP1A SGSH IL1RN DAPK1 LAMA2 MCL1 SDHB UGT1A9 HMOX2 PLA2G4C RET FUS MAPK8IP1 NGF RPS6KA3 ACE AKR1B1 HDAC6 APOA1 SOD3 ATP7A VRK2 RORA DAXX F10 MLEC COL1A1 GOSR2 MMP9 NCK2 CGRRF1 PLOD1 BCHE CYP2B6 TXNRD1 MAOB BGLAP HRAS OPA1 IGF2BP1 AGTR1 FGFR3 ALPP ROCK1 UCP2 HACD3 IL15RA CDH1 OMA1 GRIN2B FBN1 PIK3CA USE1 TRPM4 CAMLG EMC9 EMD ILVBL ACER1 EXT1 PTPN11 GDF15 MTDH VDAC1 C2CD2L MMP2 TBXAS1 THBS4 UBQLN2 SCN1A RMRP NOD2 LRPAP1 RNF19B UGT1A10 GLUD1 YWHAZ AGPAT1 RELA ALG11 LMAN1L FAAH PRKCSH UFC1 HUWE1 CBY1 ENTPD5 SORL1 TRAPPC5 CREB3L4 ATXN2L EEF1B2 DICER1 DAB2IP UFD1 GOLPH3 DYSF PPP1CA SPTLC1 DCSTAMP TLR3 TNFSF10 NR1H3 ADAMTSL1 ARFGAP2 SERPINE1 AGRN MAP3K7 LRP6 SERPINC1 KPNA2 UBE2D3 ACP1 B2M ELOVL5 G6PC2 SNAP25 TEX2 ARSA DHDDS CHEK1 ATP13A2 PXN LGALS1 BAG3 CAPN2 NAPA SSR3 PDIA5 UGT1A8 ATP5MK CAMK2A GCH1 VMA21 NPM1 GPAT3 MYH6 PLD3 BMP2 BSG ABCC8 PSMD2 ATP2C1 F7 CRAT RPA1 PSENEN RARA NEPRO KCNQ2 SIRT2 DPM3 HLA-G IL2 PCNA EGR1 GPAA1 ABCG1 CDIPT TRAPPC9 NPPB PLPP3 AFG3L2 PDLIM1 PRKD1 HCRT PIGA RPTOR SIRT3 HSPG2 PIK3R2 HSPA6 GRP VCL YIF1B CAMK2D PCK1 DAD1 SLC6A1 DNAJA1 STAT1 COPG1 ANKLE2 CYP17A1 SLC1A1 ITGB1 TREM2 ACTG1 VPS33A SLC37A1 KCNMA1 FBXO6 ABCA1 SELENOT NOX5 TLR2 PARP16 COL13A1 BID LAMP2 GPX8 PLA2G4A PPARA LGI4 YBX1 MSRA UGT1A7 CACNA1A UQCRFS1 FLT3 RAP1GDS1 WWOX ARSH PIGC ALDH3A2 UGT1A4 NACA ALPL MSRB1 COLGALT1 SLC27A2 PIGB CUL3 UGT1A3 PMEL YTHDF2 TRAPPC4 TRA LPL KMT2B SLC4A1 UBQLN4 ATP1A1 TRPV1 SETD2 PRKRA CYP19A1 LGALS3 POFUT1 YOD1 TAOK3 PTPRC MIR34A DLAT RPE65 GFAP LRIT3 FAM120A TRAF6 EPHX1 UCHL1 UVRAG PLP1 CDK2 SI PARG UBE2D2 CCDC88A SLC8A3 SCN10A EOGT EIF5A NPC2 KCNN4 AGT ELOVL2 ANXA2 PIGH NSFL1C TM7SF2 DPM2 XIAP TH PIGS BAD PSMA7 RACK1 HTR1A USP13 VAMP1 PNKD WDR83OS USP9X H2AX ADAMTS13 UGT1A CASP1 FITM2 MET USP14 ARF1 PPIA ASL SEC22A IL18 PTPA RASGRF2 TGFA PPIF TRAP1 LRRC59 HSPA1L KL RAB3GAP1 NRG1 FASLG CYP2A6 TBL2 PMAIP1 CYP2C9 CDKAL1 HP PEMT VKORC1L1 HTR3A MEF2C PDHB ELOVL7 NDUFS4 HSPA2 COPB2 CDKN2A ATG9A TFEB TRAPPC12 ACSL3 CSNK2A1 CHP1 TRPM8 UGT1A5 TTF2 IAPP PNPLA6 PURA FLNB CERS6 ARRB1 NF1 TESPA1 HNRNPU HLA-DRB1 MAN2B1 KCNB1 LIPC RUVBL2 CFLAR PALS1 SMPD2 RPL10 SPCS2 SMN1 NCLN SERPINI1 HADHB CDH2 NIBAN1 UBE2N AGPAT2 GFPT1 MYRF BRAF PRKDC ULBP1 PRKAB1 LIN28A RIC3 DRD1 DUOXA1 ANKS4B HSD11B2 OLR1 KCNIP4 MEF2A LMNB1 CASR SEC22C SERPINA2 SELP EIF2B1 PEX11B AVP PLCG1 PDE5A ACBD3 QDPR CR1 TIAL1 ZDHHC4 EEF1D TPM1 TMEM67 CHRM3 LPCAT1 ATF1 PTK2 GOLGB1 CSTB LDHA CASC3 ERBB2 ATG7 CLN5 NCSTN SYNCRIP PLG NUP210 SGF29 YY1 ELOVL1 MUC5AC NRAS SLC9A1 OPRM1 HNF4A NSDHL STEEP1 FGF2 TOR1AIP1 MIR199A1 AIMP1 PEF1 STARD5 TRPC1 DLG1 EZH2 CDKN1B CYP21A2 MYLK E2F1 SRL KRT8 TLR7 CTH BLZF1 GH1 POMP RPLP0 PTP4A1 IGF1R STXBP1 OPRD1 UBE2D1 TMEM199 NR4A1 TJP1 RPS6 GABRA1 EPAS1 TRAM2 NDUFS8 DGAT2 SPAG5 TSC1 CCK UBXN2B NPPA SLC2A4 DEGS1 DYNC1H1 GTF2I ELOVL3 PROS1 MATN3 FGFR4 HLA-DPB1 ENPP1 DYRK1A CCL4 BIRC2 DHX36 F3 CD59 DELE1 MECP2 STK39 LRP2 KRTCAP2 ABCC6 RAF1 NAT8 PREP C6orf120 DDHD1 GCLC C3orf52 CYP51A1 HACE1 MIR21 VDR PRKCQ LSG1 CCN2 GPI PGRMC1 LAMP1 SPP1 FGF21 ATXN3 RPS3 MYO9A TGFBR1 CAST ACSF3 ACER3

1. WGCNA
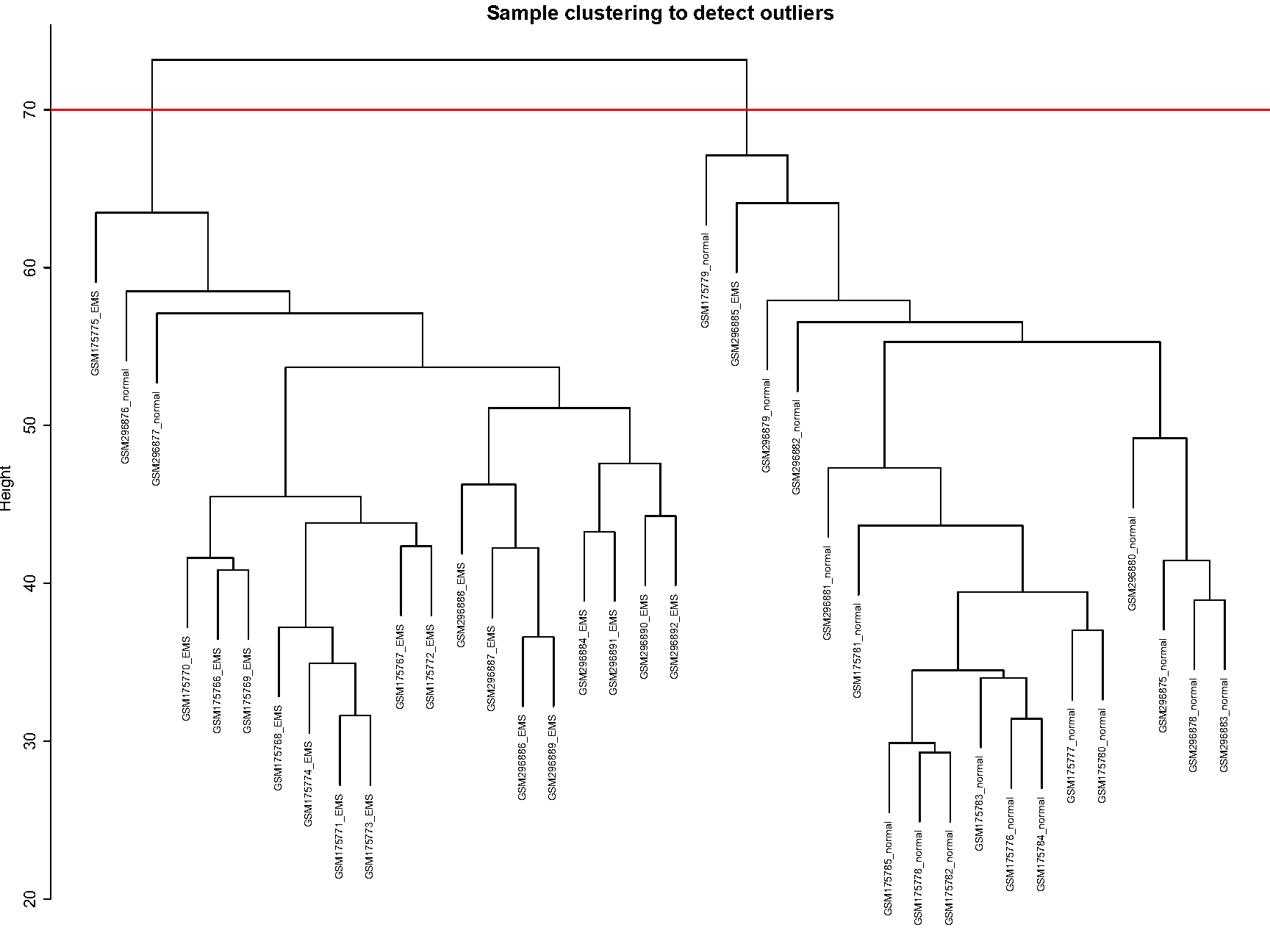


Sample cluster tree


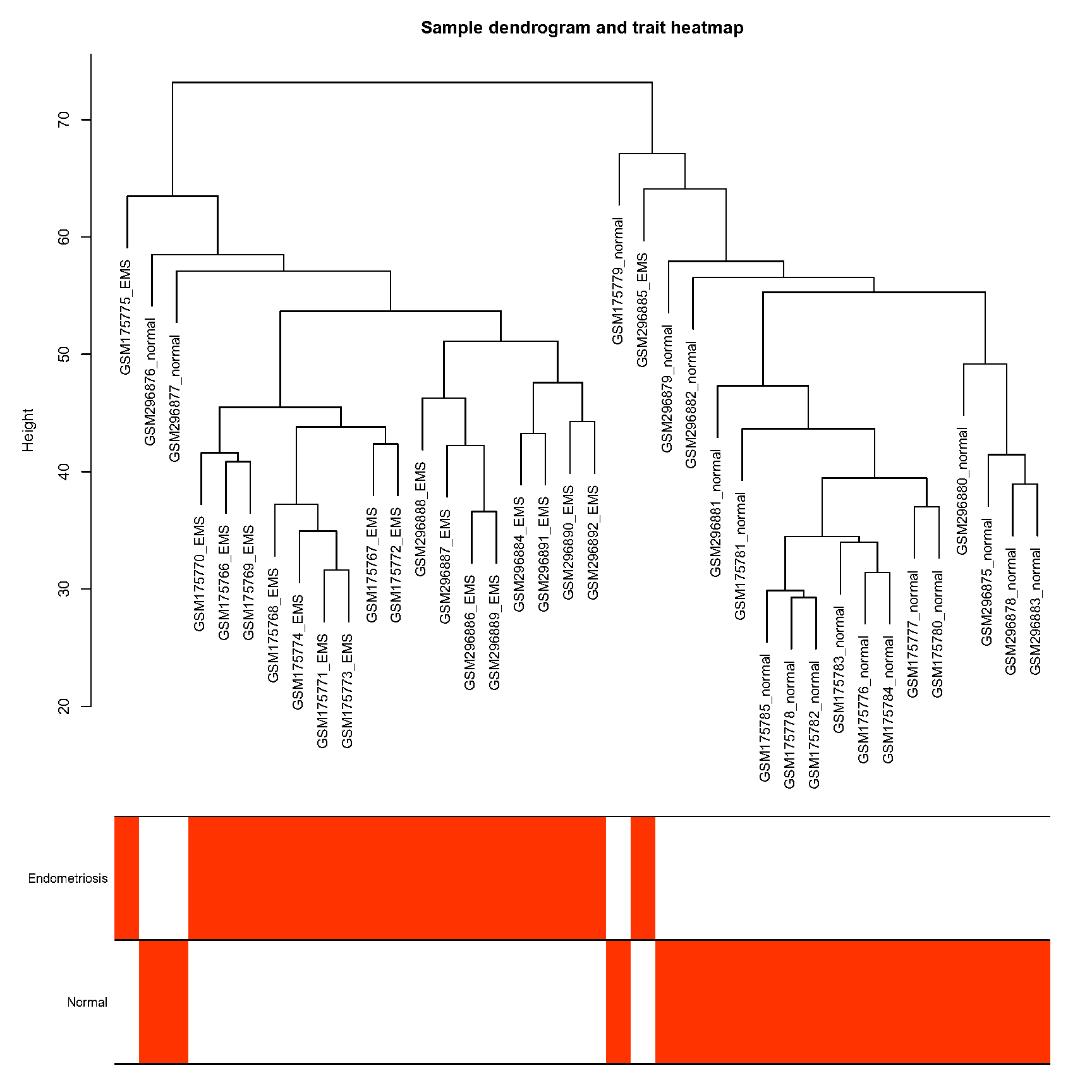


Sample heatmap


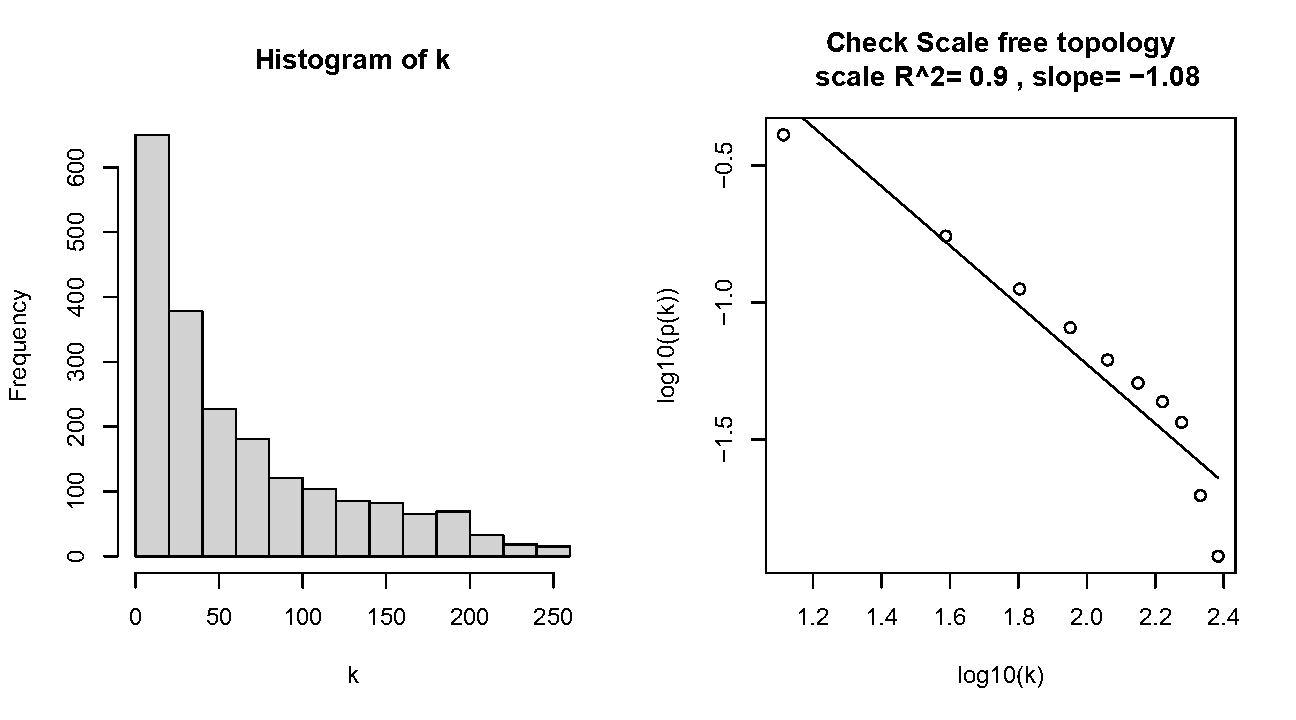


Examine whether the memory network approximates scale free for selected values of β

It can be seen that k is negatively correlated with p(k) (correlation coefficient 0.9), indicating that the chosen value of β is able to build a gene scale-free network.


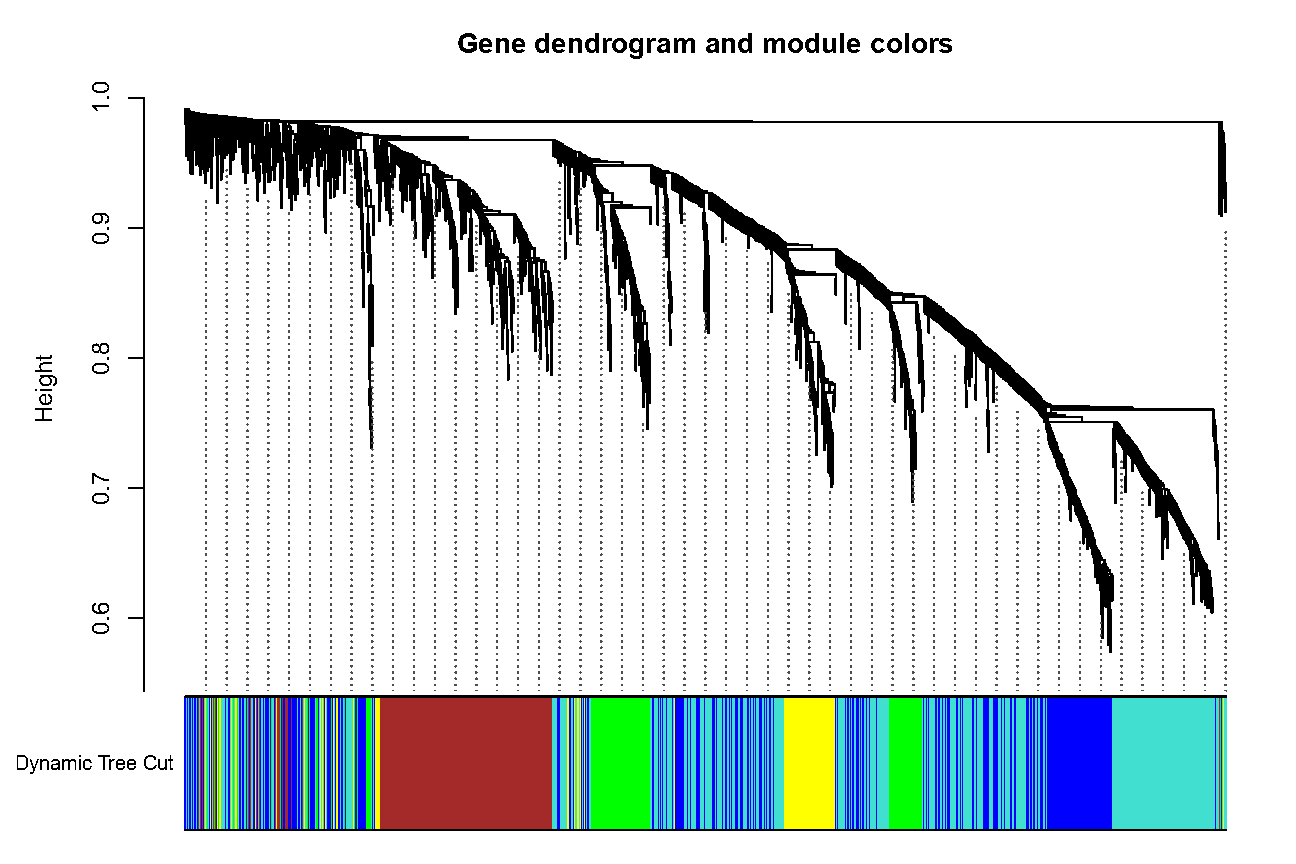


Module clustering map after merging similar modules

1. Cluster DEGs

VCAM1 GIMAP6 EPAS1 LCP2 EOMES KLRC3 THBD ITM2A FAM241A IGFBP3 SERPINA5 KCNK3 APOBEC3G KIR3DL2 NID2 KIR2DL4 LMOD1 GIMAP4 KDR GATA6 GIMAP8 SCIN COTL1 EDNRB SPINK2 GPR137B SH2D2A AEBP1 ELMO1 KIR3DL3 ZAP70 TGM2 GCH1 GREM2 TNFAIP8 KIR3DL1 NNMT KIR3DS1 CD247 LINC00996 STK26 CD2 SERPING1 GPNMB SHISAL1 KIR2DL3 IGFBP1 PTGIS SOD2 ITGAL TOX3 EVI2A PGRMC1 HCLS1 RBP4 PGM5-AS1 CCR1 ASRGL1 ARHGAP30 ST3GAL1 LAMB3 COMP HABP2 FCER1G GPR34 GXYLT2 SLC6A12 FBXO32 KIR2DS4 ENO2 SLC22A4 FAM169A SNX10 PIK3R6 LEFTY2 GEM ARSL UNC5CL CYS1 ITGAD FGR MTARC1 ARSB ETNK1 DNER CFL2 RGS16 PRRG1 PCCA-DT KLRB1 S100A4 DNAJC6 CD96 TTC39A THBS2 CYTIP LACTB2 KIR2DS2 HSD11B2 RHEX BCL2A1 BDKRB2 ELOVL7 G0S2 STYK1 P3H2 ACKR1 PCDH17 PLAAT4 SPOCK1 MAP2K6 STXBP6 RAP2C SLC7A7 LURAP1L KIAA1755 CXCL14 KIR2DS5 DOCK8 RIPOR3 EDN3 NREP S100A3 ARHGAP9 MMP26 PSTPIP1 TMEM97 CNTN4 ANKRD55 TIMP3 IL10RA TNNC1 CHST11 PRIMA1 CD300A EFEMP1 SORD PTPRR NACC2 NRG2 SST MAMDC2 WDR77 PROS1 LOC100129098 TMEM140 FOXO1 GZMB GPX3 PDGFA CD55 PRL PRICKLE1 CRYAB NUPR1 RUNX3 GPBAR1 AMIGO2 RSPO3 SERPINE2 IL2RB C3 BCL6 IRS2 ANKRD35 PIK3CD TRPC4 GNG4 GGTA1 STX18 APOL4 ADAM19 GADD45A FGL2 PARVA ADCY1 CDA LYPD3 PTGR1 GUCY1B1 TAGLN KIR2DS1 PDZK1IP1 STUM TMEM252 PLCH1 IRX5 EFNA1 ABCC3 PDPN SLC38A1 SLCO4A1 MLPH C11orf96 FAM20A CHRDL1 DPH3 PRR15 ATP2C2 KIR2DL2 CD3E NOS3 NDRG1 CDH3 KIFC1 MOGAT1 TSPAN8 EVI2B RASGRP1 TUNAR PIK3R3 CCL4 PLA2G2A GPC4 CCL2 CNN1 RAC2 TGFBR3 LIF RNASE4 CCDC3 TMOD1 LINC02349 NCAPG C12orf75 RIPPLY3 MPPED2 FBLN5 RNF19B ASS1 LMCD1 FAM83D C4BPA ANG PKMYT1 DEPP1 SLC2A3 CDYL2 EMILIN3 CORO1C KIAA1210 ENPEP STARD5 VTCN1 EFHD1 TRNP1 IGF1 IL15RA TRDC CAV1 NKG7 AOC3 HEY2 RAMP3 TMEM132C C2CD4A SASH3 MAP3K8 SPSB1 TRDV3 SYBU CDCA8 HSPB7 SPDEF ELP3 PABPC4L PRR15L FBLN2 SLC15A1 CALB2 IRX3 MMRN1 TUBA4A IRF1 MUC20 CCNB2 ITGA3 IL4R CFB SVIL BICD1 MFAP4 CCDC146 CCL21 MGP CYB5A CYFIP2 LAMA4 ZNF281 DHRS3 GZMK S100A1 CCEPR ALPL LINC00645 MUC16 DDX52 CYP3A5 SHMT1 SLA OFD1 TYMP RASSF2 GZMA IL1B GPR183 TMEM150C CCNB1 IGFBP6 TLE2 PNP IL1R2 HELLS FGB MYBL2 KIF20A ST6GALNAC1 AQP3 HOXA11 NFIL3 CSRP2 RIMKLB KAZALD1 SLITRK6 BATF3 COLEC11 SOX17 ALDH8A1 NEXN SLC7A4 LCN12 NOX4 PAEP CYP24A1 SPINK1 DRAIC CPA3 ATP1B1 SPP1 PYGM FNDC5 SRARP CDK1 C1orf116 TENT5B FXYD4 RBP7 PPP1R14C LINGO2 CSDC2 DUSP5 ALOX5AP PBK KIF4A FERMT1 TCN1 PTCH1 CITED4 EIF4E3 GNLY HEY1 IL2RG CST7 PRUNE2 HPGD IGSF9 GBP2 NEK2 TMEM45B PVRIG CORO1A PLD6 ICAM1 PLEKHG4B HPS3 SFRP4 PIP5K1B NDRG2 GAST DOK2 EPHB3 RBMX CDT1 CCL20 MYL9 SLC12A8 SELENOM ARHGAP45 PGR ZBTB8A PARVG SYNPO GJB6 TTK GGT5 PCDH20 ADAMTS16 NUSAP1 FOXC1 CTSW TNFRSF18 SOX7 IER3 CCDC170 RARRES1 SELPLG INSYN1 SAPCD2 SFMBT2 ANKRD52 TPX2 OIP5 LOC100505912 HPSE DPYSL3 MIR22HG LRRTM1 OLFM1 PKP2 FLRT1 LOC101928419 PRKAR2B FARS2-AS1 ZWINT GALNT12 ECI2 CRISPLD1 RAD54B DLGAP5 FAR2P2 SOCS3 LRRC3B TOP2A OVGP1 CD7 PSRC1 MND1 GLT1D1 KIF11 UBE2T PRC1 MOCOS SLC15A2 MYRF TNFAIP6 KRT23 HGF MST1R PRF1 FLRT2 CHI3L1

4. **List of abbreviations**

AUC Area Under Curve

BP Biological Processes

CC Cellular Component

EMS Endometriosis

ERS Endoplasmic reticulum stress

GEO Gene Expression Omnibus

GO Gene Ontology

GS Gene Significance

GSEA Gene set enrichment analysis

KEGG Kyoto Encyclopedia of Genes and Genomes

LASSO Least Absolute Shrinkage and Selection Operator

MCC Maximum Clique Centrality

MF Molecular Function

MM Module Membership

PCA Principal Component Analysis

PPI Protein-Protein Interaction networks

RF Random Forest

RMSE Root Mean Squared Error

ROC Receiver Operating Characteristic

SVM-RFE Support Vector Machine-Recursive Feature Elimination

WGCNA Weighted Correlation Network Analysis

1. XGBoost Result


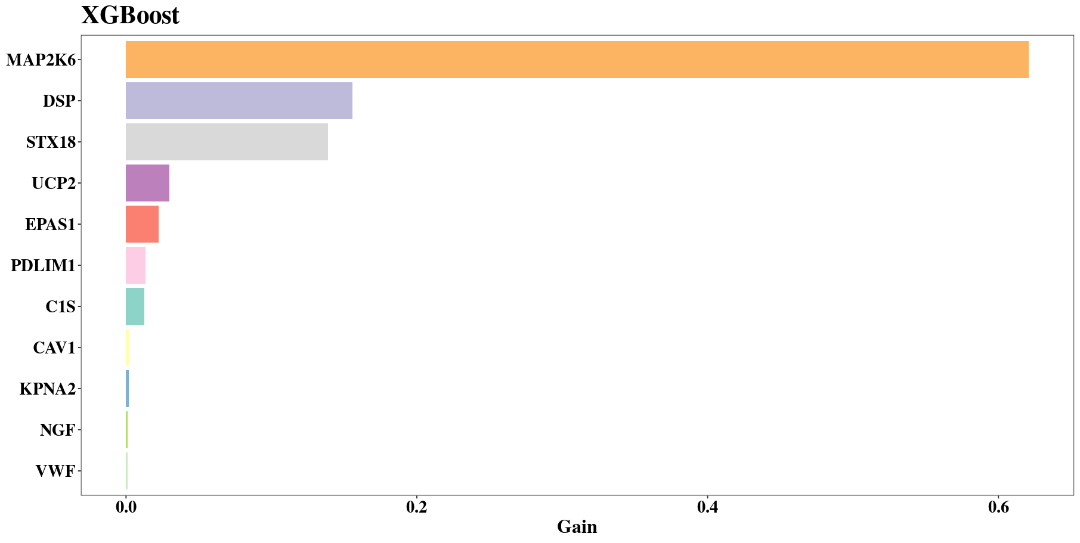


1. Four machine learning intersection


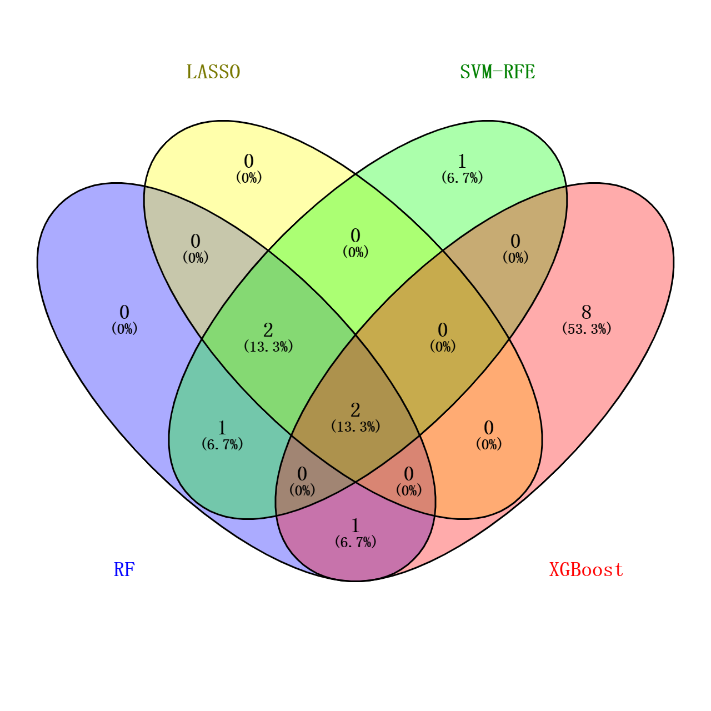

Supplement: Supplementary file 1 — Supplementary Information 1. [file 41598_2025_22400_MOESM1_ESM.docx]
